# Non-Canonical Cannabinoid Receptors with Distinct Binding and Signaling Properties in Prostate and Other Cancer Cell Types Mediate Cell Death

**DOI:** 10.3390/ijms23063049

**Published:** 2022-03-11

**Authors:** Amal M. Shoeib, Lance N. Benson, Shengyu Mu, Lee Ann MacMillan-Crow, Paul L. Prather

**Affiliations:** Department of Pharmacology and Toxicology, College of Medicine, University of Arkansas for Medical Sciences, Little Rock, AR 72205, USA; amshoeib@uams.edu (A.M.S.); lnbenson@uams.edu (L.N.B.); smu@uams.edu (S.M.); macmillancrowleea@uams.edu (L.A.M.-C.)

**Keywords:** cannabinoid receptors, non-canonical, prostate cancer, cytotoxicity, mitochondria, WIN-55,212-2

## Abstract

Cannabinoids exert anti-cancer actions; however, the underlying cytotoxic mechanisms and the cannabinoid receptors (CBRs) involved remain unclear. In this study, CBRs were characterized in several cancer cell lines. Radioligand binding screens surprisingly revealed specific binding only for the non-selective cannabinoid [^3^H]WIN-55,212-2, and not [^3^H]CP-55,940, indicating that the expressed CBRs exhibit atypical binding properties. Furthermore, [^3^H]WIN-55,212-2 bound to a single site in all cancer cells with high affinity and varying densities. CBR characteristics were next compared between human prostate cancer cell lines expressing low (PC-3) and high (DU-145) CBR density. Although mRNA for canonical CBRs was detected in both cell lines, only 5 out of 15 compounds with known high affinity for canonical CBRs displaced [^3^H]WIN-55,212-2 binding. Functional assays further established that CBRs in prostate cancer cells exhibit distinct signaling properties relative to canonical G_i_/G_o_-coupled CBRs. Prostate cancer cells chronically exposed to both CBR agonists and antagonists/inverse agonists produced receptor downregulation, inconsistent with actions at canonical CBRs. Treatment of DU-145 cells with CBR ligands increased LDH-release, decreased ATP-dependent cell viability, and produced mitochondrial membrane potential depolarization. In summary, several cancer cell lines express CBRs with binding and signaling profiles dissimilar to canonical CBRs. Drugs selectively targeting these atypical CBRs might exhibit improved anti-cancer properties.

## 1. Introduction

Cancer is the second most common cause of death following heart disease in the US and it is estimated that over 600,000 patients in the U.S. will die of cancer in 2021 [1]. Most malignancies, including prostate cancer, pheochromocytoma, and glioma, are often treated with a combination of chemotherapeutic agents and radiotherapy [2,3,4]. Although such multi-agent chemotherapeutic regimens represent the predominant treatment of many cancers, this approach is often associated with severe side effects, subsequent co-morbidities, and complications [4,5,6]. For instance, docetaxel is a mainstay chemotherapeutic agent employed to manage prostate cancer; however, its use results in a host of adverse events, such as muscle weakness, GI distress, neuropathies, fluid retention, and pneumonitis [7,8]. Radiotherapy used to treat prostate cancer, glioma, and pheochromocytoma is also associated with severe side effects, including deterioration of neurocognitive functions, endocrine dysfunction, insomnia, and fatigue [3,5,9]. Furthermore, androgen deprivation therapy for management of prostate cancer often results in osteoporosis, muscle weakness, hot flashes [10], and increased risk of diabetes and cardiovascular disease [11]. Therefore, alternative therapeutic options are needed that efficaciously inhibit tumor growth, while resulting in fewer of the deleterious adverse effects that often accompany current multi-regimen chemotherapy and radiotherapy associated with treatment of most cancers.

Preclinical studies investigating the anti-cancer effects of endogenous and synthetic phytocannabinoids have been extensively reviewed [12,13]. For example, treatment of colon cancer cells with CBR agonists, such as Δ^9^-tetrahydrocannabinol (THC) and JWH-133, results in apoptosis and reduces invasiveness and migration to other regions by enhancing tumor necrosis factor-alpha (TNFα)-induced ceramide synthesis, regulating ER stress-related genes and diminishing angiogenesis [14,15]. Moreover, cannabidiol (CBD) augments the cytotoxic effects of anti-cancer drugs for treatment of head and neck squamous cell carcinoma via apoptosis and autophagy [16]. A recent study also showed that the synthetic cannabinoid WIN-55,212-2 exerts anti-proliferative actions in human glioma cells through reactive oxygen species (ROS) induction [17].

Even though published studies over the past several decades have provided valuable insight into the potential use of cannabinoids for reduction of cancer cell growth and metastasis, there are still many gaps in current knowledge. First, evidence remains inconclusive regarding whether cannabinoids mediate anti-tumor effects directly through CBRs [18] or via CBR-independent mechanisms [19]. Second, in most studies to date, CBRs are identified in cancer cells primarily by use of only quantitative real-time polymerase chain reaction (qRT-PCR) and Western blots [20,21], while complete characterization of binding and functional properties of expressed CBRs is lacking. Third, it is debated whether CBR density contributes to cancer prognosis and/or treatment outcome. For example, some studies indicate that CBR overexpression is protective and leads to desirable therapeutic outcomes in cancer treatment [22], while other studies demonstrate a direct correlation between CBR upregulation in cancer with poor prognosis and that downregulation of CBR expression by agonists actually decreases proliferation [23,24,25]. Finally, CBRs expressed in cancer cells have been shown to form heteromers with other GPCRs that exhibit distinct pharmacological characteristics [26,27,28,29] and, upon activation, reduce tumor cell growth [30,31,32] and cell migration [27]. Therefore, to aid in the development of more efficacious cannabinoid-derived anti-cancer agents, studies designed to better understand of the role of CBRs in mediating cancer cell death are needed.

Our laboratory recently reported that TC-71 and A-673 Ewing sarcoma (EWS) cell lines (a form of pediatric bone cancer) express high levels of non-canonical CBRs that exhibit distinct binding and signaling profiles, and ligands binding with high affinity to these receptors produce cytotoxicity and cell death [33]. In the present study, it was hypothesized that other cancer types similarly express non-canonical CBRs and that drugs targeting these receptors will impact tumor cell growth and proliferation. Therefore, a variety of cancer cell lines, including C6-glioma (mouse), PC-12 pheochromocytoma (rat), HeLa cervical cancer (human), and human prostate cancer cell lines (PC-3 and DU-145) were screened for the presence of non-canonical CBRs. Subsequent studies focused on the characterization of signaling properties of CBRs expressed in PC-3 and DU-145 cells and whether treatment of DU-145 cells (expressing the highest density of CBRs) with compounds exhibiting high affinity for non-canonical CBRs would affect receptor density, cell viability, and mitochondrial integrity.

## 2. Results

### 2.1. Competitive Receptor Binding: CBRs Expressed in a Variety of Cancer Cell Types Exhibit Atypical Binding Properties Relative to Canonical CBRs

**Figure 1:**
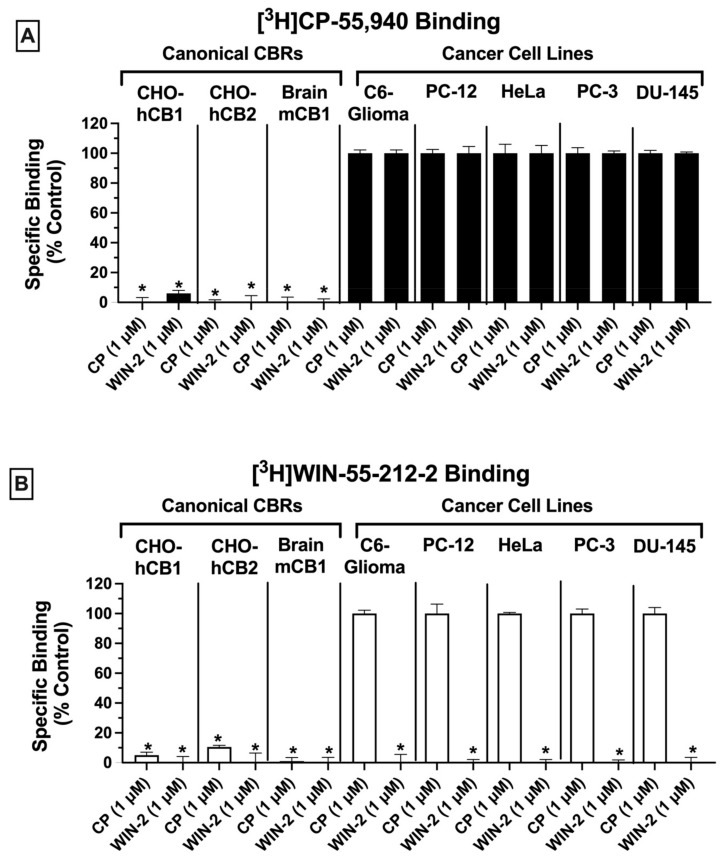
Competitive receptor binding: CBRs expressed in a variety of cancer cell types exhibit atypical binding properties relative to canonical CBRs. Competitive binding screens were conducted by using the well-characterized non-selective CB1/CB2 radioligands [^3^H]CP-55,940 (0.2 nM) (**A**) or [^3^H]WIN-55,212-2 (1 nM) (**B**). Bars represent the amount of radioactivity remaining when radioligands were co-incubated with a high, receptor-saturating concentration (1 μM) of non-radioactive CP-55,940 (left bars) or a second non-selective CBR ligand WIN-55,212-2 (right bars) in all cell lines/tissues. The cell lines/tissues screened were CHO-hCB1 (expressing canonical human CB1Rs), CHO-hCB2 (expressing canonical human CB2Rs), mouse brain (expressing canonical mouse CB1Rs), and cancer cell lines C6-glioma (mouse glioma), PC-12 (rat pheochromocytoma), HeLa (human cervical carcinoma), PC-3 (human prostate cancer), and DU-145 (human prostate cancer). In cells expressing canonical CBRs, specific binding could be observed by using both radioligands, displaced by both non-radioactive cannabinoids. In marked contrast, specific binding in cancer cells could only be observed when using [^3^H]WIN-55,212-2, which could only be displaced by non-radioactive WIN-55,212-2 but not CP-55,940. Bars represents the means ± SEM of data collected from a minimum of three experiments. Significantly different from 100% (one-sample *t*-test, * *p* < 0.05).

Radioligand binding screens were first used to determine whether CBRs were expressed in mouse (C6-glioma), rat (PC-12), and human (HeLa, PC-3 and DU-145) cancer cell lines (Figure 1). Initially, binding screens were conducted by using the well-characterized non-selective CB1/CB2 radioligand [^3^H]CP-55,940 (Figure 1A). Specifically, the ability of a high, receptor-saturating concentration (1 μM) of non-radioactive CP-55,940 or a second non-selective CBR ligand WIN-55,212-2, to displace [^3^H]CP-55,940 (0.2 nM) was examined (Figure 1A). Since the affinity of both CP-55,940 and WIN-55,212-2 for canonical CB1 and CB2 receptors is high (e.g., in the low nM range), under these conditions the receptor-saturating concentration (1 μM) of these non-radioactive drugs used for screening should produce complete displacement of the tracer concentration (0.2 nM) of the radioligand used ([^3^H]CP-55,940) if CBRs are present.

As expected, in CHO cells stably expressing either canonical human CB1 or CB2 receptors (CHO-hCB1 and CHO-hCB2, respectively) or in membranes prepared from mouse brain containing a high density of mouse CB1 receptors (mCB1), both CP-55,940, and WIN-55,212-2 (1 μM) produced near or complete displacement of [^3^H]CP-55,940 (Figure 1A; left 3 panels). The binding data presented for CHO-hCB1 and CHO-hCB2 cells have been previously reported by our laboratory [33]. In marked contrast (and unexpectedly), when using membranes prepared from all cancer cell lines examined (C6-glioma, PC-12, HeLa, PC-3, and DU-145), neither the non-selective CBR ligand CP-55,940 nor WIN-55,212-2 produced significant displacement of [^3^H]CP-55,940 (Figure 1A; right 5 panels). Although no specific binding was observed by displacement of [^3^H]CP-55,940 (0.2 nM) when co-incubated with 1 μM of CP-55,940, the presence of non-specific binding sites would likely have been identified if higher concentrations of CP-55,940 to displace [^3^H]CP-55,940 were examined. Therefore, before definitive conclusions can be made concerning the use of [^3^H]CP-55,940 to define CBR populations in these cancer cells, future experiments fully characterizing [^3^H]CP-55,940 binding properties in membranes prepared from cancer cells relative to transfected cells or brain tissue will be required.

Similar experiments were next conducted by employing a second well-characterized CB1/CB2 radioligand [^3^H]WIN-55,212-2 (1 nM; Figure 1B). As observed with results obtained using [^3^H]CP-55,940 (and as expected), in cells/tissues expressing canonical CB1 and CB2 receptors, both CP-55,940 and WIN-55,212-2 (1 μM) produced near or complete displacement of [^3^H]CP-55,940 (Figure 1B; left 3 panels). As indicated in Figure 1A, the binding data presented for CHO-hCB1 and CHO-hCB2 cells have been previously reported by us [33]. However, and strikingly distinct, when examining binding in various cancer cells (C6-glioma, PC-12, HeLa, PC-3, and DU-145), only WIN-55,212-2, and not CP-55,940, significantly displaced [^3^H]CP-55,940 (Figure 1B; right 5 panels).

Taken together, the results from these binding screens strongly indicate that CBRs expressed in a variety of cancer cell types exhibit atypical binding properties relative to canonical CBRs, with specific binding being detected using [^3^H]WIN-55-212-2 but not [^3^H]CP-55,940. The presence of potential “non-canonical” CBRs in cancer cell lines suggested by these results is similar to observations reported previously by our group for CBRs expressed in Ewing sarcoma cell lines [33].

### 2.2. Homologous Receptor Binding: Cancer Cell Lines Express Non-Canonical CBRs in Varying Densities (B_MAX_)

**Figure 2:**
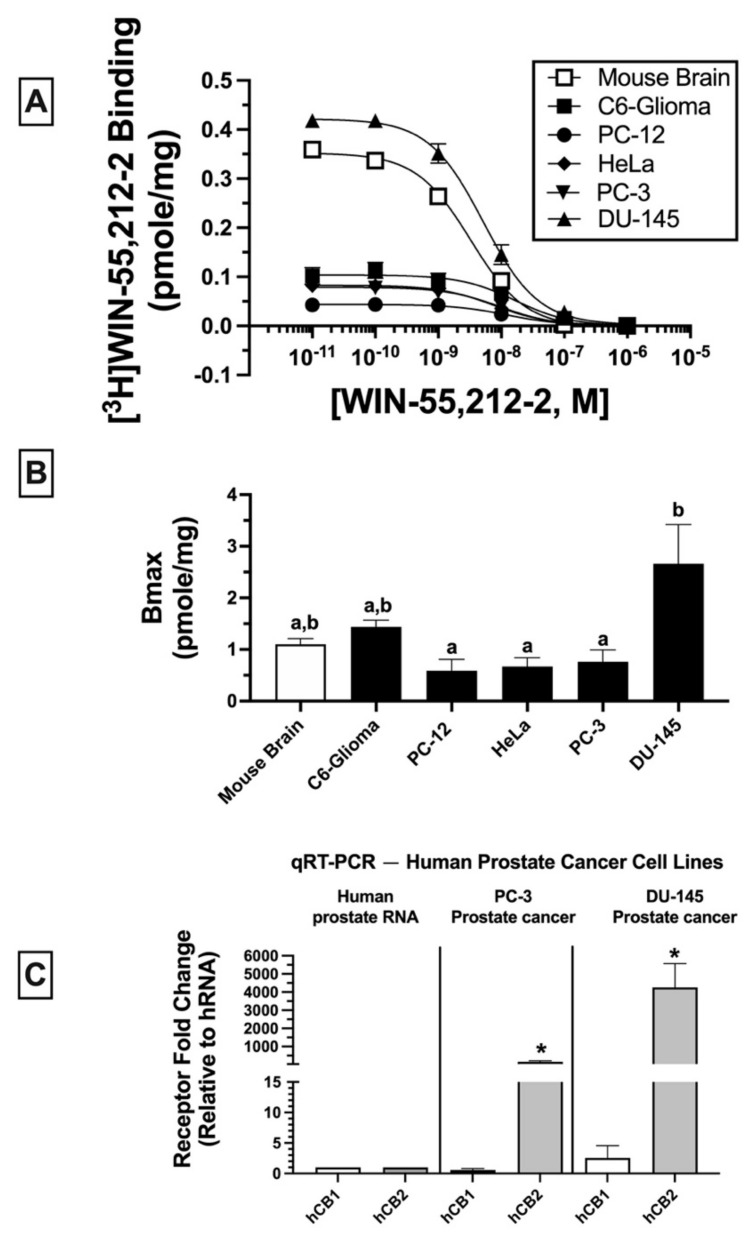
Homologous receptor binding: cancer cell lines express non-canonical CBRs in varying densities (B_MAX_). (**A**) Homologous receptor binding studies demonstrated that WIN-55,212-2 produced concentration-dependent (10^−11^ to 10^−6^ M) displacement of [^3^H]-WIN-55,212-2 (1 nM) binding, a one-site model fitting best. Data for each concentration represent the mean ± SEM of data collected from a minimum of three experiments, with each data point conducted in triplicate for each experiment. (**B**) Receptor density (B_MAX_) was compared across cancer cell lines and mouse brain via non-linear regression curve-fitting utilizing a one-site homologous competition binding equation to determine K_D_ and B_MAX_ values. ^a,b^ Bars designated with different letters are significantly different from each other (one-way ANOVA followed by Tukey’s post hoc test for multiple comparisons, *p* < 0.05). (**C**) qRT-PCR was used to examine mRNA levels for canonical human CB1 and CB2Rs. Data are presented as fold changes in mRNA levels in either PC-3 (**middle** panel) or DU-145 (**right** panel) cells compared to human prostate mRNA control values that were assigned an arbitrary value of 1 (**left** panel). Significantly different from human prostate mRNA control (one-way ANOVA followed by Dunnett’s post hoc test, * *p* < 0.05).

Homologous binding assays using [^3^H]WIN-55-212-2 were subsequently conducted to assess the affinity (K_D_) of WIN-55,212-2 for non-canonical CBRs expressed in the cancer cell lines examined (Figure 2A) and to determine the density (B_MAX_) of the receptors expressed (Figure 2B).

In all membrane homogenates examined, WIN-55-212-2 produced a concentration-dependent decrease in [^3^H]WIN-55,212-2 (1 nM) binding (Figure 2A). Non-linear regression analysis revealed that all competition binding data were best fitted by a one-site model. WIN-55-212-2 exhibited high affinity (K_D_) in the nM range for expressed receptors (e.g., 4.3–16.1 nM) but did not vary significantly across cancer cell lines examined (C6-glioma: 16.1 ± 2.5 nM; PC-12: 12.6 ± 2.7 nM; HeLa: 7 ± 1.6 nM; PC-3: 8.4 ± 2.1; DU-145: 4.3 ± 0.89 nM). However, WIN-55,212-2 exhibited slightly higher affinity for canonical CB1Rs expressed in mouse brain (2.2 ± 0.02 nM) relative to all cancer cell lines tested.

When comparing receptor density (B_MAX_) across cancer cells lines (Figure 2B), DU-145 cells expressed a significantly higher CBR density (2.66 ± 0.76 pmole/mg) relative to PC-12 (0.59 ± 0.22 pmole/mg), HeLa (0.67 ± 0.17 pmole/mg), and PC-3 (0.76 ± 0.23 pmole/mg) cells, but not when compared to mouse brain (1.1 ± 0.11 pmole/mg) or C6-glioma (1.44 ± 0.13 pmole/mg) cells.

Collectively, homologous receptor binding studies suggest that WIN-55,212-2 binds to a single site with similar high affinity for non-canonical CBRs differentially expressed in all cancer cell lines examined, although with slightly lower affinity than for canonical CB1Rs in mouse brain. Moreover, CBRs in cancer cells are expressed in densities (B_MAX_) comparable to high levels of CBRs endogenously occurring in mouse brains.

### 2.3. Quantitative Real-Time PCR (qRT-PCR): Human Prostate Tissue and Human Prostate Cancer Cell Lines Express mRNA for Canonical CB1 and CB2 Receptors

Due to atypical binding properties described for CBRs expressed in cancer cell lines thus far, qRT-PCR was next employed to determine whether mRNA for canonical CB1 and/or CB2 receptors was detectable in cancer cells (Figure 2C). To focus the study, all subsequent experiments were limited to investigation of human prostate cancer lines expressing a low (PC-3) and high (DU-145) density of non-canonical CBRs (see Figure 2B). qRT-PCR experiments revealed that normal (non-cancerous) human prostate tissue expresses mRNA for canonical CB1 and CB2 receptors (Figure 2C; left panel); however (and interestingly), mRNA for CB2 (but not CB1) receptors is dramatically upregulated in PC-3 (160-fold; Figure 2C, middle panel) and DU-145 (over 4000-fold; Figure 2C, right panel) cells when compared to basal expression levels in normal human prostate tissue. The higher CB2 receptor mRNA expression in DU-145 compared to PC-3 cells is in agreement with the higher density (B_MAX_) of CBRs determined by homologous receptor binding studies shown previously (Figure 2B).

Collectively, these findings indicate that although CBRs in human prostate cancer cells lines PC-3 and DU-145 exhibit atypical binding properties, these cells nevertheless express mRNA for canonical CB1 and CB2 receptors, with CB2R mRNA being dramatically upregulated in both cancer cell lines relative to normal prostate tissue.

### 2.4. Competitive Binding Screens: CBRs Expressed in PC-3 and DU-145 Prostate Cancer Cell Lines Exhibit Atypical Binding Profiles for Many Well-Characterized CBR Ligands Relative to Canonical CBRs

**Figure 3:**
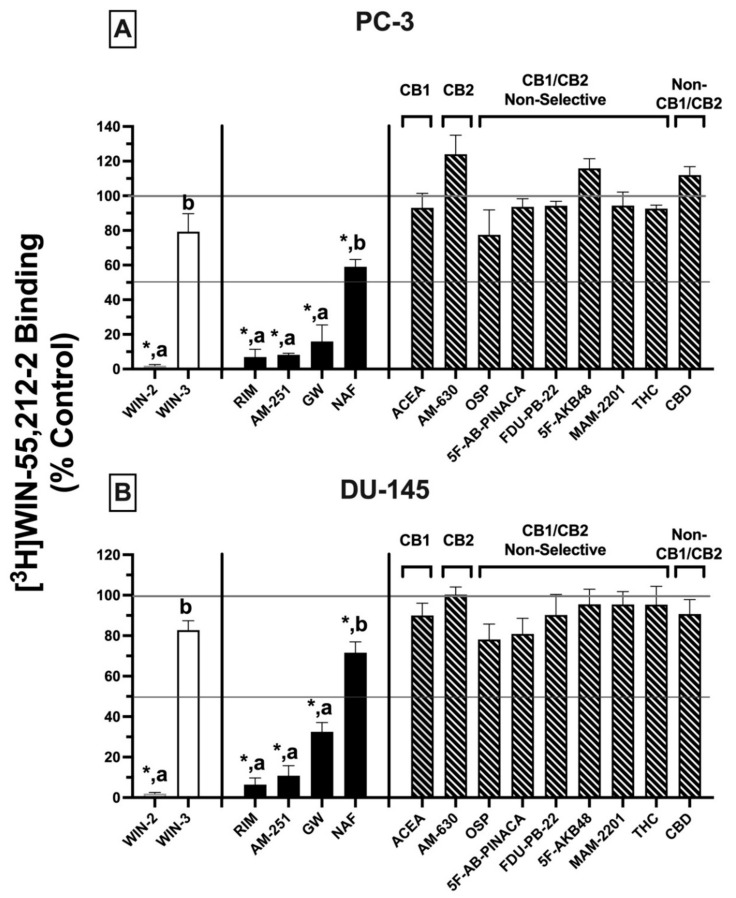
Competitive binding screens: CBRs expressed in PC-3 and DU-145 prostate cancer cell lines exhibit atypical binding profiles for many well-characterized CBR ligands relative to canonical CBRs. A variety of cannabinoids with varying selectivity for canonical CBRs were tested (1 μM) for their ability to displace [^3^H]WIN-55,212-2 (1 nM) from CBRs expressed in PC-3 (**A**) and DU-145 (**B**) membranes. Bars represents the means ± SEM of data collected from a minimum of three experiments. Significantly different from 100% (one-sample *t*-test, * *p* < 0.05). ^a,b^ Compounds designated with different letters are significantly different from each other (one-way ANOVA followed by Tukey’s post hoc test for multiple comparisons, *p* < 0.05).

Initial receptor binding screens revealed that CBRs expressed in several cancer cell lines exhibited atypical binding properties because specific binding could only be observed when employing [^3^H]WIN-55,212-2 but not [^3^H]CP-55-940, and only non-radioactive WIN-55,212-2 (but not CP-55,940) could compete for [^3^H]WIN-55,212-2 binding to expressed CBRs (Figure 1). To further characterize CBRs expressed in PC-3 and DU-145 cell lines, experiments were next designed to investigate the binding pattern of a number of structurally diverse synthetic phytocannabinoids known to exhibit high affinity for canonical CB1 and/or CB2 receptors (Figure 3)_._

For these experiments, because all drugs examined exhibit high nM affinity for canonical CBRs, a receptor-saturating concentration (1 μM) was employed for all presented screening experiments (Figure 3A,B). Similar to results presented in Figure 1B, non-radioactive WIN-55,212-2 (1 μM) produced complete displacement of [^3^H]WIN-55,212-2 (1 nM) from CBRs expressed in both DU-145 and PC-3 cells. In marked contrast, WIN-55,212-3 (1 μM) (a stereoisomer of WIN-55,212-2 [34]) resulted in only 20.7 ± 10.3% (PC-3; Figure 3A) and 13.9 ± 2.7% (DU-145; Figure 3B) displacement of [^3^H]WIN-55,212-2 (1 nM) from expressed CBRs. Such stereoselective binding is characteristic of ligands interacting with G protein-coupled receptors (including CBRs), providing additional support that CBRs expressed in prostate cancer cells that bind WIN-55,212-2 are likely G protein-linked.

In addition to WIN-55,212-2, as anticipated, several other compounds (1 μM) with high nM affinity and differential selectivity for canonical CB1Rs (rimonabant, AM-251) or CB2Rs (GW-405833) resulted in greater than 50% displacement of [^3^H]WIN-55,212-2 (1 nM) from CBRs expressed in both PC-3 (Figure 3A) and DU-145 (Figure 3B) cells. Under the conditions used for this assay, compounds producing displacement over 50% would be predicted to exhibit high affinity (K_i_ values) in the nM range [35]. In addition to these well-characterized CBR ligands, a selective estrogen receptor modulator (SERM), nafoxidine, previously demonstrated by our laboratory to exhibit high affinity for both canonical CBRs [36], also produced significant (but less) displacement of [^3^H]WIN-55,212-2 in both cell lines. Very surprisingly (if CBRs expressed were canonical), several well-established CB1-selective (ACEA), CB2R-selective (AM-630), or CB1/CB2R non-selective synthetic (OSP, 5F-AB-PINACA, FDU-PB-22, 5F-AKB48, MAM-2201) phytocannabinoids (THC, CBD) tested failed to displace [^3^H]WIN-55,212-2 (1 nM) from PC-3 (Figure 3A) or DU-145 (Figure 3B) membranes.

Collectively, the atypical binding profiles in human prostate cancer cells presented for many well-characterized CBR ligands relative to those predicted for canonical CBRs provide additional support for our initial binding screens (Figure 1), suggesting that the CBRs expressed in the cancer cell lines examined here are indeed non-canonical. Importantly, it is also noteworthy that atypical binding patterns of compounds observed were almost identical in both PC-3 and DU-145 cells, suggesting that expression of a common receptor in both cell lines is likely.

### 2.5. Competitive Binding Screens: CBRs Expressed in PC-3 and DU-145 Prostate Cancer Cell Lines Are Not Transient Receptor Potential (TRP) Channels

In addition to CBRs, WIN-55,212-2 has been shown to also modulate activity of several TRP channels, generally requiring relatively high μM concentrations [37]. Since the affinity of WIN-55,212-2 for non-canonical receptors in cancer cells reported here is in the low nM range, it is unlikely that the receptors characterized here are TRP channels. However, to examine this possibility, similar receptor binding screens were conducted to determine whether several TRP channel ligands (1 μM) could displace [^3^H]WIN-55,212-2 (1 nM) in DU-145 membranes. Neither the high affinity TRPV1 ligands capsaicin [38], PDDHV [39], and CAY-100448 [40] nor the TRPA1 ligand HC-030031 [41] produced significant displacement of [^3^H]WIN-55,212-2 (data not shown). Although TRPV3 and TRPV4 ligands N-oleoyl-valine [42] and HC-067047 [43], respectively, did produce 15 ± 2.7 and 22 ± 3.8% displacement, these results would predict very low μM affinity for the expressed non-canonical receptors, which is inconsistent with the high nM affinity these ligands are known to exhibit for TRP channels [38,39,40,41,42,43]. These results suggest that non-canonical cannabinoid receptors expressed in DU-145 cells are not TRP channels.

### 2.6. Competition Receptor Binding Curves: Three Cannabinoids Exhibit High nM Affinity (K_i_) for Non-Canonical CBRs Expressed in Prostate Cancer Cell Membranes

**Figure 4:**
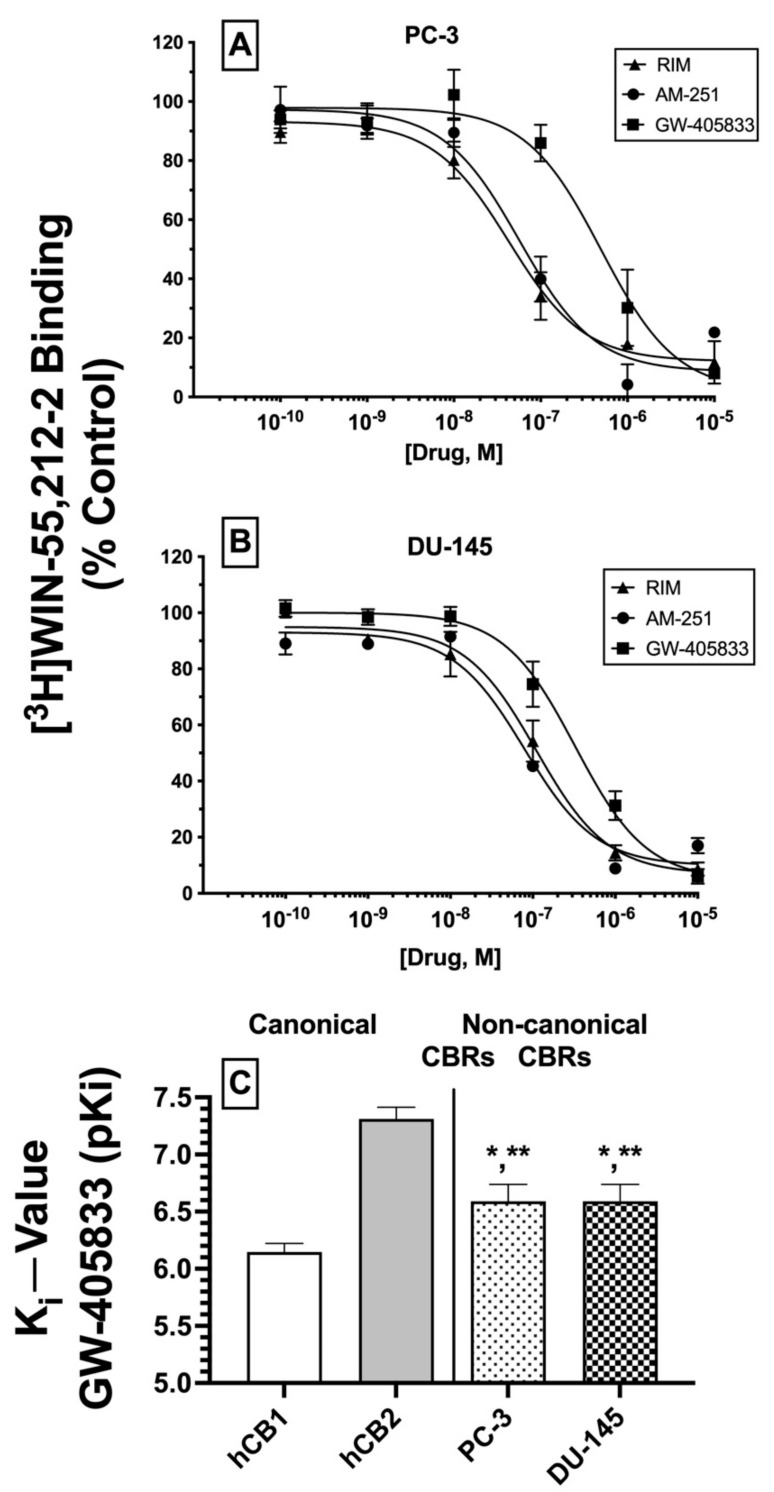
Competition receptor binding curves: three cannabinoids exhibit high nM affinity (K_i_) for non-canonical CBRs expressed in prostate cancer cell membranes. Increasing concentrations (10^−10^ to 10^−5^ M) of cannabinoid ligands producing the greatest displacement of [^3^H]WIN-55,212-2 (as shown in Figure 4A,B; rimonabant, AM-251 and GW-405833), were co-incubated with [^3^H]WIN-55,212-2 (1 nM) in PC-3 (**A**) and DU-1453 (**B**) membranes. (**C**) Affinity (K_i_ values) of GW-405833 was compared between canonical CBRs expressed in CHO-hCB1 and CHO-hCB2 cells (**left** panel) and CBRs expressed in PC-3 and DU-145 membranes (**right** panel). IC_50_ values derived from binding curves converted to K_i_ values via the Cheng–Prusoff equation [35] (Table 1). Data for each concentration represent the mean ± SEM of data collected from a minimum of three experiments, with each data point conducted in triplicate for each experiment. Significantly different from K_i_ values for canonical hCB1 and hCB2 receptors, respectively (unpaired *t*-test, * *p* < 0.05, ** *p* < 0.001).

Based on the initial competitive binding screens using only a single 1 μM concentration (Figure 3A,B), we next employed full competition receptor binding curves to determine the actual affinity (K_i_) of the three cannabinoid ligands that produced greater than 50% displacement of [^3^H]WIN-55,212-2 in both PC-3 (Figure 4A) and DU-145 (Figure 4B) membranes. As predicted from the initial screens, both CB1-selective antagonists/inverse agonists rimonabant and AM-251 were found to exhibit high nM affinity (K_i_ values of 100 nM or less) for CBRs expressed in PC-3 and DU-145 membranes (Table 1). Moreover, the CB2-selective agonist GW-405833 also bound to CBRs in PC-3 and DU-145 cells with slightly lower affinity when compared to rimonabant and AM-251, but still with a high nM affinity of approximately 300 nM (Table 1).

Next, it was determined whether the affinity of GW-405833 for CBRs expressed in prostate cancer cells differed from the affinity of this CB2-selective compound for canonical CBRs. As such, K_i_ values of GW-405833 were compared between human CB1 and CB2 receptors in membranes prepared from CHO cells stably expressing each type of canonical CBR and CBRs expressed in PC-3 and DU-145 cells (Figure 4C). To provide a normal distribution of data for statistical analysis, K_i_ values were converted to pK_i_ (−log K_i_) values. As anticipated for a CB2-selective ligand [44], GW-405833 bound with higher affinity to human CB2 (K_i_ = 51 nM) compared to human CB1 (K_i_ = 747 nM) receptors. Interestingly, although GW-405833 bound with similar affinity to CBRs expressed in both PC-3 and DU-145 membranes (~290 nM), this affinity was significantly different from that determined for binding to either canonical CB1 or CB2 receptors expressed in CHO cells.

An alternative interpretation of these competition receptor binding experiments might be that the curves presented may not result from competition of these ligands with [^3^H]WIN-55,212-2 at a single receptor but instead represent a composite curve, consisting of [^3^H]WIN-55,212-2 competition for binding to both canonical CB1 and CB2 receptors present in the membranes examined. While this interpretation cannot be conclusively ruled out, it is unlikely given that all three ligands examined exhibit relatively selective binding to CB1 (e.g., rimonabant and AM-251) or CB2 (e.g., GW-405833) receptors. Therefore, if these ligands were binding to both CB1 and CB2 receptors to produce a composite curve reflected as a “sum of competition of [^3^H]WIN-55,212-2 at these two receptors”, due to a near or greater than 100-fold difference in ligand affinity for CB1 and CB2 receptors, the resultant curves would have been predicted to be bi-phasic. In contrast, all curves presented were best fitted by a one-site model when analyzed by non-linear curve fitting. In any case, when all receptor binding data are taken as a whole, it is relatively clear that CBRs expressed in the cancer cells examined exhibit very distinct receptor binding and functional properties relative to canonical cannabinoid receptors.

In summary, as predicted from initial (1 μM) binding screens, three cannabinoid ligands do indeed bind with high nM affinity to CBRs expressed in PC-3 and DU-145 cells, with similar rank order of affinity in both prostate cancer cell lines. Additionally, GW-405833 binds to CBRs expressed in prostate cancers with an affinity that is significantly different from the affinity of this CB2-selective agonist for either human canonical CB1 or CB2 receptors. Taken together, these data provide additional support that CBRs expressed in prostate cancer cells differ significantly from canonical CB1 and CB2 receptors.

### 2.7. CBRs Expressed in Prostate Cancer Cells Exhibit Distinct Signaling Properties Relative to Canonical G_i_/G_o_-Coupled CBRs

Following characterization of the binding properties of non-canonical CBRs expressed in PC-3 and DU-145 cells, subsequent experiments were carried out to define and compare the signaling pathways of these receptors (e.g., G protein activation and adenylyl cyclase modulation) with those of canonical CBRs.

#### 2.7.1. G Protein Activation ([^35^S]GTPγS Binding)

**Figure 5:**
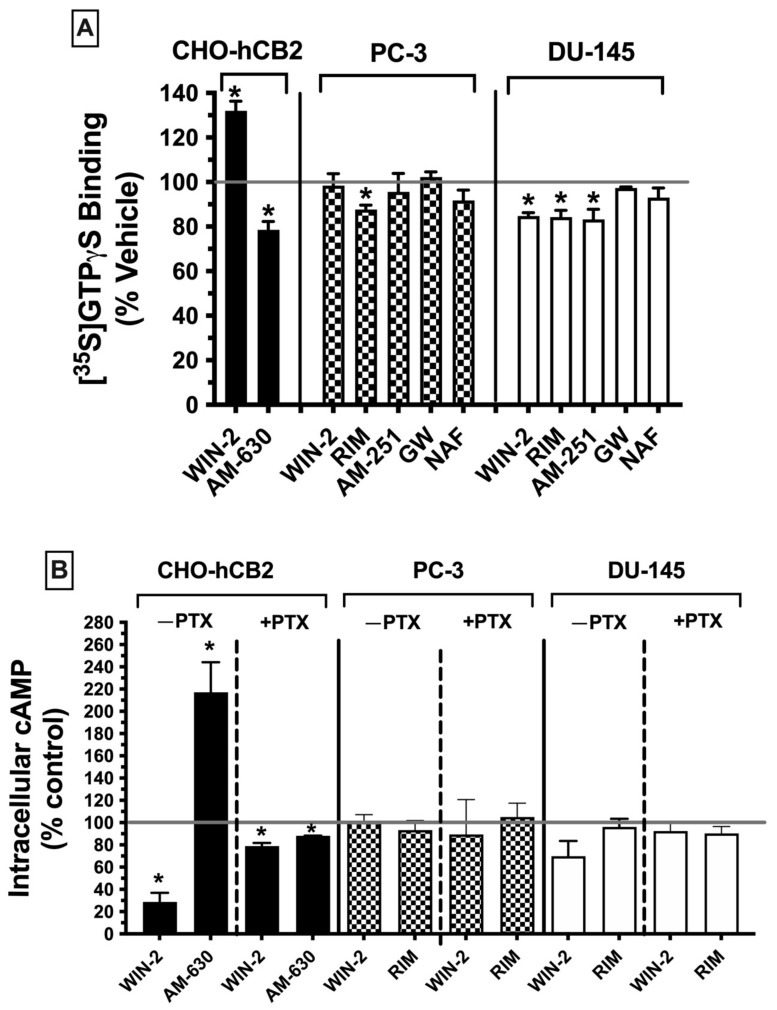
CBRs expressed in prostate cancer cells exhibit distinct signaling properties relative to canonical G_i_/G_o_-coupled CBRs. (**A**) Regulation of G protein activity by canonical CBRs stably expressed in CHO-hCB2 (**left** panel), PC-3 (**middle** panel), and DU-145 (**right** panel) membranes was assessed. Specifically, changes in [^35^S]GTPγS binding produced by incubation of respective membranes with a receptor-saturating concentration (10 μM) of different CBR ligands were quantified. (**B**) Modulation of adenylyl cyclase activity in whole cells by canonical CBRs stably expressed in CHO-hCB2 (**left** panel), PC-3 (**middle** panel), and DU-145 (**right** panel) cells was assessed. Specifically, changes in forskolin-stimulated intracellular [^3^H]cAMP levels produced by incubation of respective membranes with a receptor-saturating concentration (10 μM) of different CBR ligands were quantified. Bars for both [^35^S]GTPγS binding and cAMP levels represent the means ± SEM of data collected from a minimum of three experiments and are presented as the percentage of basal activity (100%). Significantly different from basal activity (* *p* < 0.05, one-sample *t*-test from 100%).

Canonical CBRs are G protein-coupled receptors (GPCRs), with effects primarily linked to activation of the G_i/o_ subtype of G proteins [45]. Activation of CBRs by agonists results in an increase of G_i/o_ protein activity, whereas antagonists have no effect, and because CBRs are constitutively active receptors [46], inverse agonists decrease basal G protein signaling. G protein activity of G_i/o_-linked receptors can be quantified in membrane homogenates by measuring [^35^S]GTPγS binding in the presence of agonists. As such, these experiments were conducted to determine whether CBRs expressed in prostate cancer cells are G_i/o_ protein-coupled, by measuring the ability of high-affinity ligands to modulate G protein activity, the first step in the signaling cascade (Figure 5A).

Initially, [^35^S]GTPγS binding activity was assessed using CHO-hCB2 homogenates to serve as a positive control, demonstrating results to be expected by well-established CBR ligands for modulation of G protein signaling via canonical CBRs. As anticipated, a receptor-saturating concentration of the CB1/CB2 receptor full agonist WIN-55,212-2 (10 μM) significantly increased [^35^S]GTPγS binding in CHO-hCB2 homogenates by 32.0 ± 4. 28%, whereas the CB2R inverse agonist AM-630 (10 μM) decreased basal G protein activity by 26.15 ± 5.15% (Figure 5A; left panel). In marked contrast, when G protein activity was examined in membranes prepared from PC-3 (Figure 5A; middle panel) and DU-145 cells (Figure 5A; right panel), neither the agonist at canonical CB1/CB2 receptors WIN-55-212-2 (10 μM) nor the CB2-selective agonist GW-405-833 (10 μM) increased [^35^S]GTPγS binding. In fact, WIN-55,212-2 significantly decreased [^35^S]GTPγS binding below basal levels (by 15.3 ± 1.6%) in DU-145 membranes (Figure 5A; right panel), as might be expected for an inverse agonist. However, similar to action at canonical CBRs, the CB1 antagonists/inverse agonists rimonabant (in both PC-3 and DU-145) and AM-251 (only in DU-145) did reduce [^35^S]GTPγS binding below basal levels. Finally, the CB1/CB2 receptor antagonist/inverse agonist nafoxidine had no effect on G protein activity, indicative of its being a neutral antagonist. Therefore, the functional profile for G protein modulation of CBRs expressed in both prostate cancer cell lines is clearly distinct from canonical CBRs, most notably shown by a lack of agonist activity produced by the full CB1/CB2 agonist WIN-55.212-2 and full CB2-selective agonist GW-405-833.

#### 2.7.2. Adenylyl Cyclase Modulation in Whole Cells

CBRs are G protein-coupled receptors that activate G_i/o_ proteins, which subsequently go on to inhibit the intracellular effector adenylyl cyclase, resulting in a decrease in intracellular cAMP levels [47]. Accordingly, CBR agonists (via increasing G_i/o_ protein activity) reduce cAMP levels, whereas neutral antagonists produce no effect and inverse agonists (via inhibition of constitutively active CBRs) increase cAMP levels. To examine the next step in the CBR intracellular signaling cascade after G protein activation in prostate cancer cells, modulation of the activity of the downstream enzyme adenylyl cyclase was evaluated by quantifying intracellular forskolin-stimulated cAMP levels in the presence of a receptor-saturating concentration (10 μM) of high-affinity CBR ligands (Figure 5B).

First, to serve as a positive control for how ligands act at canonical CBRs, adenylyl cyclase activity was measured in intact whole CHO-hCB2 cells in the presence of either the full CB1/CB2R agonist WIN-55,212-2 (10 μM) or the CB2R-inverse agonist AM-630 (10 μM) (Figure 5B; left panel). As expected, WIN-55,212-2 (10 μM) reduced forskolin-stimulated intracellular cAMP levels by 71.3 ± 8.2%, whereas AM-630 (10 μM) enhanced cAMP levels by 117 ± 27.1%. Pertussis toxin produces ADP-ribosylation of the G_i/o_ protein α-subunit, resulting in the uncoupling of G proteins from GPCRs and thus eliminating signaling activity [48]. Consequently, also as anticipated for G_i/o_-linked CBRs, overnight treatment of CHO-hCB2 cells with pertussis toxin completely abolished the effects of ligands on adenylyl cyclase activity on CBRs in CHO-hCB2 cells (Figure 5B; left panel).

Next, forskolin-stimulated intracellular cAMP levels were quantified in the presence of the canonical CBR agonist WIN-55,212-2 (10 μM) or the canonical CBR antagonist/inverse agonist rimonabant (10 μM) in both PC-3 and DU-145 cells (Figure 5B; middle and right panels). Contrary to results observed in CHO-hCB2 cells expressing the canonical CB2 receptor, neither WIN-55-212-2 nor rimonabant significantly altered intracellular cAMP levels in prostate cancer cells, whether treated with pertussis toxin or not.

Collectively, results from both G protein activation and adenylyl cyclase modulation assays suggest that CBRs expressed in prostate cancer cells exhibit distinct signaling properties relative to canonical G_i_/G_o_-coupled CBRs.

### 2.8. Chronic Treatment of Cells with CBR Ligands: Prolonged Exposure of PC-3 and DU-145 Cells to Both High-Affinity CBR Agonists and Antagonists/Inverse Agonists Results in CBR Downregulation

**Figure 6:**
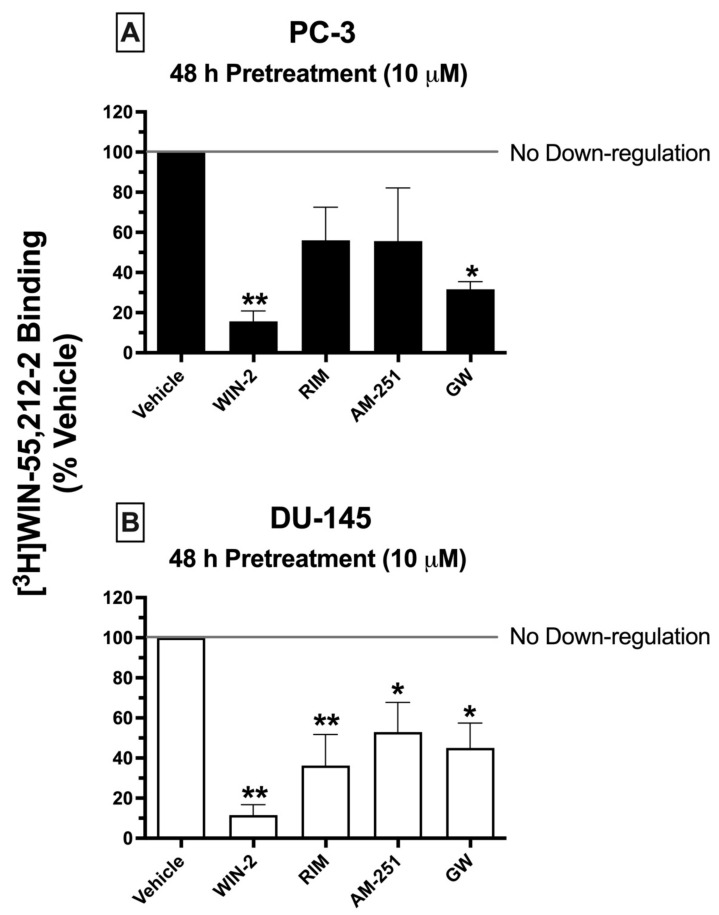
Chronic treatment of cells with CBR ligands: prolonged exposure of DU-145 and PC-3 cells to both high affinity CBR agonists and antagonists/inverse agonists results in CBR downregulation. PC-3 (**A**) and DU-145 (**B**) cells were cultured for 48 h in the presence of a receptor-saturating concentration (10 μM) of the different CBR ligands examined. Following chronic treatment, cells were thoroughly washed to remove residual drug, harvested, and cell lysates were prepared. Bars represent the amount of specific [^3^H]-WIN-55,212-2 binding (1 nM) remaining as compared to cells treated for 48 h with vehicle only and are presented as the means ± SEM of data collected from a minimum of three experiments. Significantly different from 100% (one-sample *t*-test, * *p* < 0.05, ** *p* < 0.001).

Prolonged exposure of most GPCRs (including CBRs) to agonists results in an important adaptive process, known as receptor downregulation, in which receptors are removed from the plasma membrane by internalization and eventually degraded to reduce continued stimulation [49]. To further characterize the signaling properties CBRs expressed in prostate cancer cells, experiments were next designed to determine whether chronic treatment with high-affinity CBR ligands would produce receptor downregulation in PC-3 and DU-145 cells similar to that occurring following prolonged treatment of cells expressing canonical CBRs (Figure 6).

Similar to what would be expected in cells expressing canonical CBRs, chronic exposure (48 h) of prostate cancer cells to the canonical CB1/CB2 full agonist WIN-55,212-2 (10 μM) produced pronounced downregulation in both PC-3 (Figure 6A) and DU-145 (Figure 6B) cells, reducing the amount of [^3^H]WIN-55,212-2 (1 nM) binding by 84.33 ± 5.2 and 88.45 ± 5.22%, respectively. Similarly, 2-day treatment of both prostate cancer cell lines with the CB2-selective agonist GW-405833 (10 μM) resulted in 68.33 ± 3.8 and 55 ± 12.5% downregulation of CBRs expressed in PC-3 and DU-145 cells, respectively (Figure 6A,B). Very interestingly, and not consistent with actions at canonical CBRs, both CB1-selective antagonists/inverse agonists AM-251 and rimonabant also produced significant CBR downregulation in DU-145 cells—47.05 ± 14.8 and 63.75 ± 15.5%, respectively. However, likely due to the variability of results in PC-3 cells, chronic exposure to AM-251 and rimonabant only produced a non-significant trend towards decreased receptor expression.

To summarize, although chronic treatment of PC-3 and DU-145 cells with the agonists WIN-55,212-2 and GW-405833 did produce CBR downregulation as expected for action at canonical CBRs, prolonged exposure with the antagonists/inverse agonists AM-251 and rimonabant unexpectedly also resulted in receptor downregulation, adding additional support that CBRs expressed in prostate cancer cells are indeed non-canonical.

### 2.9. LDH Cytotoxicity: Treatment of DU-145 Cells with Ligands Exhibiting High Affinity for Expressed CBRs Induces Cell Death

**Figure 7:**
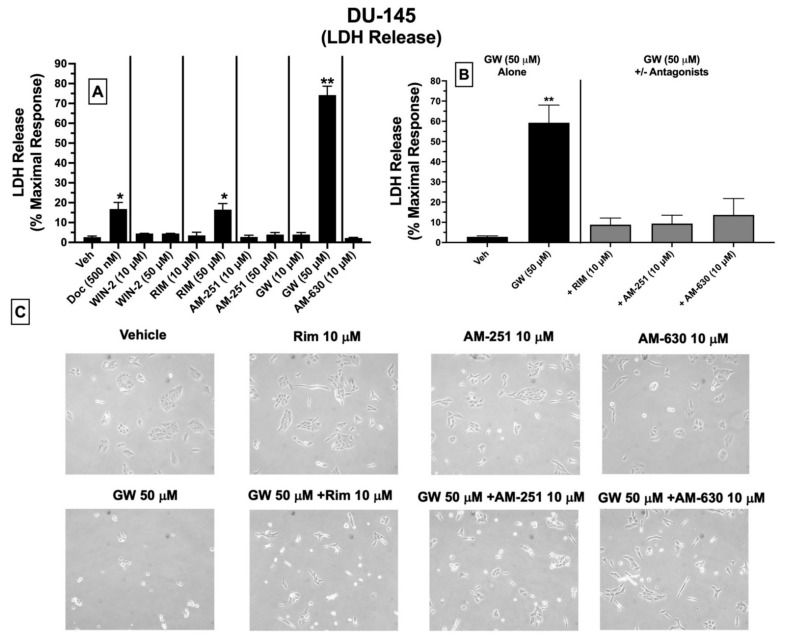
LDH cytotoxicity: DU-145 cells treated with ligands exhibiting high affinity for expressed CBRs induce cell death. Cytotoxicity was determined by assessing LDH release following 24 h incubation of DU-145 cells with CBR ligands. (**A**) LDH release was measured 24 h after treatment of cells with a lower (10 μM) and higher (50 μM) concentration of CBR ligands when administered alone. (**B**) To determine whether LDH released by GW-405833 could be antagonized by lower concentrations of CBR ligands producing little or no cytotoxicity when tested alone, LDH release was measured following co-incubation of GW-405833 (50 μM) with (10 μM of) each CBR ligand examined. (**C**) Microscopic evaluation for qualitative assessment of protection against GW-405833-induced cytotoxicity. Representative images of DU-145 cells were acquired prior to performing an LDH release assay after cells were subjected to the indicated drug treatment. Data are reported as the means ± SEM of three experiments, each conducted in triplicate (N = 3). Significantly different from 0% (one-sample *t*-test, * *p* < 0.05, ** *p* < 0.01).

Subsequent experiments were conducted to determine whether ligands with high affinity for CBRs expressed in DU-145 cells produce cytotoxicity by assessing LDH release (Figure 7). Intracellular LDH is released in growth media upon disruption of cell membranes. The proportion of released LDH from damaged/dying cells into the supernatant media can be quantified using a colorimetric kit (BioVision), where the amount of released LDH directly correlates with the extent of drug-induced cell death [50].

Quantification of LDH release showed that under the conditions employed, 24 h treatment of DU-145 cells with docetaxel (500 nM; a standard chemotherapeutic drug used to treat prostate cancer), the CB1-selective antagonist/inverse agonist rimonabant (50 μM), and the CB2-selective agonist GW-405833 (50 μM) resulted in significant increases in LDH release of 16.8 ± 3.4, 16.5 ± 3.5 and 74.2 ± 4.5%, respectively, compared to vehicle-treated cells (control) (Figure 7A). No significant effects were observed for lower doses (10 μM) of rimonabant, GW-405833, or AM-630, and neither low (10 μM) nor high (50 μM) concentrations of WIN-55,212-2, AM-251 or AM-630 altered LDH release in treated DU-145 cells (Figure 7A).

Based on a lack of LDH release produced by a lower 10 μM concentration of rimonabant, AM-251 or AM-630, it was next assessed whether coincubation with these drugs could attenuate the cytotoxic effects of GW-405833 (50 μM) in DU-145 cells (Figure 7B). Co-incubation with CB1-selective inverse agonists rimonabant and AM-251 almost completely blocked GW-405833 induced LDH release (Figure 7B). Interestingly, GW-405833-induced LDH release was also significantly attenuated upon co-incubation with AM-630, a CB2-selective antagonist/inverse agonist, which did not displace [^3^H]WIN-55,212-2 from non-canonical CBRs expressed in these cells, as shown by competition binding screens (Figure 3B). Importantly, GW-405833 produced significantly less LDH release (46.4 ± 6.4%) in an immortalized non-cancerous pancreatic cell line NPrEC [51], relative to that produced in DU-145 cells (74.2 ± 4.5%; Figure 7A).

Microscopic images were captured to qualitatively assess the effect of the tested drugs on DU-145 cellular morphology (Figure 7C). These images provide visual support for the observations that GW-405833 (50 μM) induces significant cellular stress that can be significantly attenuated by co-incubation with a concentration (10 μM) of rimonabant, AM-251 and AM-630 that has no effect on cellular morphology alone (Figure 7C).

Collectively, of all CBR ligands examined, only GW-405833 and rimonabant significantly increase LDH release (indicative of cell death) when administered alone at a relatively high concentration of 50 μM. Furthermore, the cytotoxic actions of GW-405833 may be mediated via action at a common receptor to which rimonabant, AM-251, and AM-630 bind.

### 2.10. ATP-Dependent Cell Viability: Treatment of DU-145 Cells with Ligands Exhibiting High Affinity for Expressed CBRs Reduces ATP-Dependent Cell Viability

**Figure 8:**
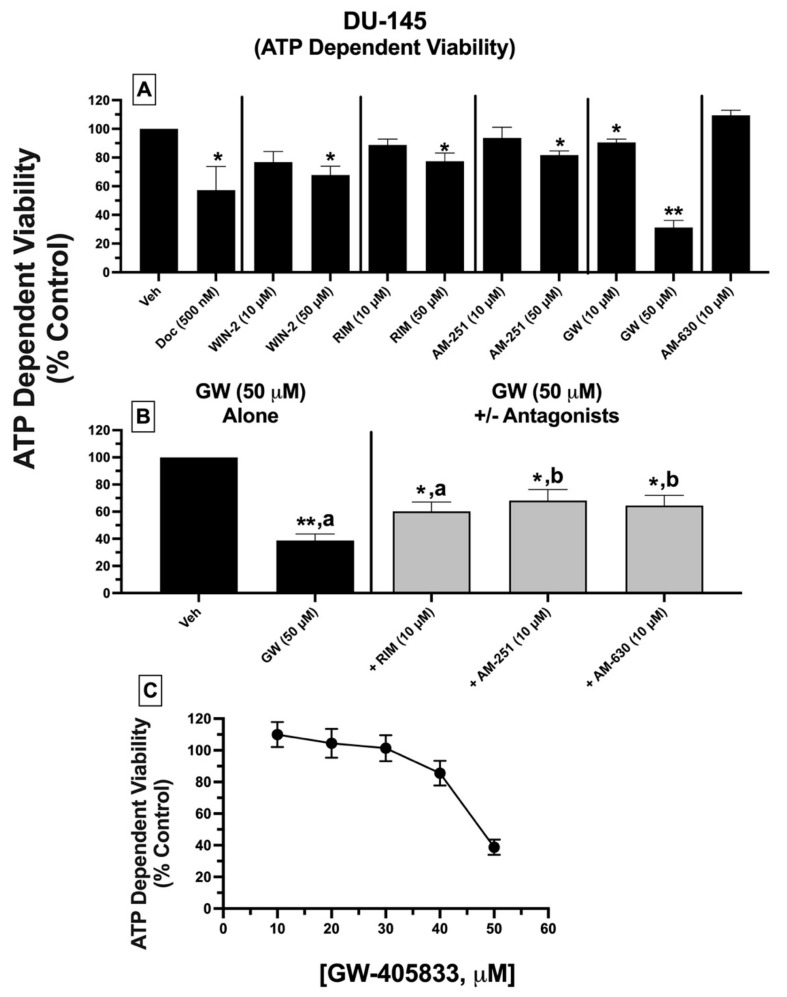
ATP-dependent cell viability: DU-145 cells treated with ligands exhibiting high affinity for expressed CBRs reduce ATP-dependent cell viability. DU-145 cell viability was determined by assessing the decrease in ATP generation following 24 h incubation of DU-145 cells with CBR ligands. (**A**) ATP concentrations were measured 24 h after treatment of cells with a lower (10 μM) and higher (50 μM) concentration of CBR ligands when administered alone. (**B**) To determine whether reductions in ATP concentrations produced by GW-405833 could be antagonized by lower concentrations of CBR ligands producing little or no effect on cell viability when tested alone, ATP levels were measured following co-incubation of GW-405833 (50 μM) with (10 μM of) each CBR ligand examined. (**C**) Experiments were conducted to determine the concentration-dependent effects of GW-405833 (10 to 50 μM, 24 h) on ATP-dependent viability in DU-145 cells. Data for each concentration represent the means ± SEM of data collected from a minimum of three experiments, with each data point conducted in triplicate for each experiment. Significantly different from 100% (one-sample *t*-test, * *p* < 0.05, ** *p* < 0.01). ^a,b^ Compounds designated with different letters are significantly different from each other (one-way ANOVA followed by Tukey’s post hoc test for multiple comparisons, *p* < 0.05).

To assess a second measure of cytotoxicity, the effect of CBR ligand treatment on the ATP-dependent cell viability of DU-145 cells was determined (Figure 8). ATP concentration is indicative of the extent of metabolic activity occurring in viable cells and directly correlates with the number of viable cells in culture [52].

DU-145 cells were treated with either vehicle, docetaxel (500 nM), or CBR ligands (10 and 50 μM), with ATP levels quantified 24 h post-treatment. Receptor-saturating concentrations (10 and 50 μM) were selected for all test compounds to predict maximal drug efficacy. DU-145 cell treatment with all CBR ligands alone (except GW-405833) at 10 μM concentrations failed to reduce ATP levels (Figure 8A). However, when drugs were tested at a higher concentration (50 μM), all significantly reduced ATP levels from 18–32%. Strikingly, and similar to effects observed for LDH release, GW-405833 (50 μM) produced the greatest reduction in ATP-dependent cell viability of 68.8 ± 4.95%.

As was examined for LDH release, subsequent experiments determined whether reduction in ATP-dependent cell viability, produced by GW-405833 (50 μM), could be antagonized by a low concentration (10 μM) of AM-251, rimonabant, and AM-630 that had no effect on ATP levels when tested alone (Figure 8B). Co-incubation with CB1-selective inverse agonist AM-251, but not rimonabant, significantly attenuated reductions in ATP concentrations produced by GW-405833 (Figure 8B). Moreover, as was observed for LDH release, GW-405833-induced reduction in ATP levels was significantly attenuated upon co-incubation with AM-630, a CB2-selective antagonist/inverse agonist, which did not displace [^3^H]WIN-55,212-2 from non-canonical CBRs expressed in these cells.

Finally, due to the high degree of ATP reduction produced, experiments were conducted to assess whether the effect of GW-405833 on DU-145 cell viability is concentration-dependent (Figure 8C). Curiously, when examining increasing concentrations ranging from 10–50 μM, GW-405833 only produced ATP-dependent reductions in cell viability at the highest 50 μM concentration. Due to the extremely steep concentration–effect curve, the potency (e.g., IC_50_ value) for GW-405833 could not be determined.

When effects on LDH release and ATP-dependent cell viability are considered together, these data collectively suggest that high-affinity CBR ligands produce significant cell death of DU-145 prostate cancer cells, mediated via a common receptor.

### 2.11. Mitochondrial Membrane Potential (MMP): Treatment of DU-145 Cells with Ligands Exhibiting High Affinity for Expressed CBRs Induces Significant Mitochondrial Depolarization, Indicative of Disruption to Mitochondrial Function

**Figure 9:**
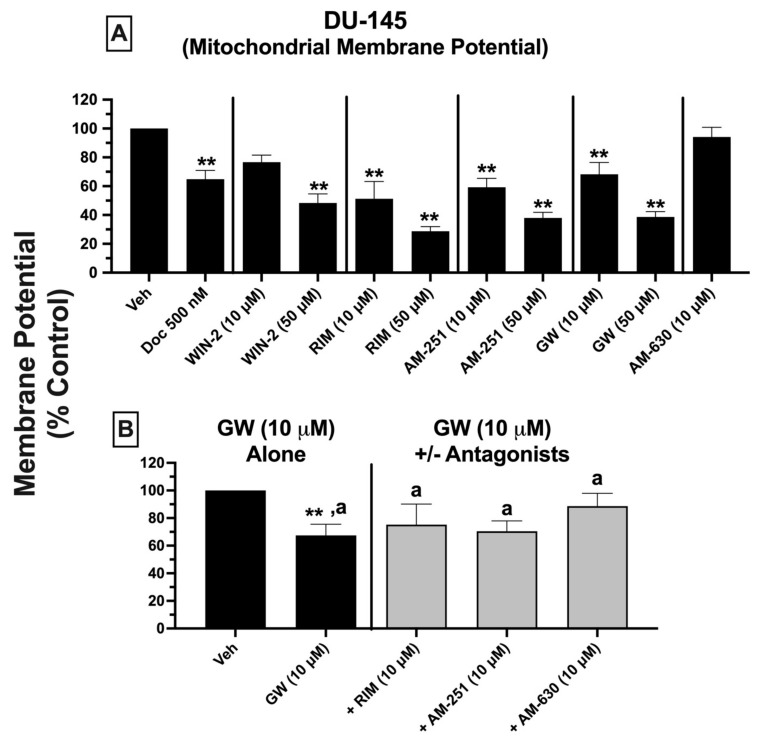
Mitochondrial membrane potential (MMP): ligands exhibiting high affinity for expressed CBRs in DU-145 cells induce significant mitochondrial depolarization, indicative of disruption in mitochondrial function. Mitochondrial membrane potential (MMP) was assessed using JC-1 staining and reading the plate using a SpectraMax M2e microplate reader following 24 h incubation of DU-145 cells with CBR ligands. (**A**) MMP was measured 24 h after treatment of cells with a lower (10 μM) and higher (50 μM) concentration of CBR ligands when administered alone. (**B**) To determine whether reductions in MMP produced by GW-405833 could be antagonized by lower concentrations of CBR ligands producing little or no MMP reduction when tested alone, the effect of MMP was measured following co-incubation of GW-405833 (10 μM) with (10 μM of) each CBR ligand examined. Significantly different from 100% (one-sample *t*-test, ** *p* < 0.01). ^a^ Compounds designated with different letters are significantly different from each other (one-way ANOVA followed by Tukey’s post hoc test for multiple comparisons, *p* < 0.05).

To investigate whether CBR ligands induce mitochondrial injury, mitochondrial membrane potential (MMP) was examined using 5,5′,6,6′-tetrachloro-1,1′,3, 3′-tetraethylbenzimidazol-carbocyanine iodide (JC-1) staining (Figure 9) [53].

DU-145 cells were treated for 24 h with either vehicle, docetaxel (500 nM, as a positive control), or low (10 μM) and high concentrations (50 μM) of all test compounds alone (Figure 9A). Docetaxel and all CBR ligands exhibiting high-affinity CBRs expressed in DU-145 cells induced significant mitochondrial membrane depolarization (as indicated by a shift from red to green fluorescence) in a concentration-dependent manner, at both 10 and 50 μM concentrations, except for WIN-55,212-2, which produced effects only at 50 μM. MMP was reduced by 40 to as much as 80%. As with previous measures of cytotoxicity (LDH release and ATP-dependent cell viability), the CB2-selective antagonist/inverse agonist AM-630, which did not displace [^3^H]WIN-55,212-2 from non-canonical CBRs expressed in these cells, also had no effect on MMP in DU-145 cells.

Since GW-405833 was the most efficacious compound in reducing DU-145 cell viability, as evident in both LDH and ATP activity assays, additional experiments were conducted to assess the effect of co-incubation with lower concentrations (10 μM) of rimonabant, AM-251, and AM-630 on the depolarizing action of GW-405833 (10 μM) on DU-145 cells (Figure 9B). When GW-405833 (10 μM) was co-incubated with all three CBR ligands, MMP was not significantly reduced from vehicle-treated levels, which suggests that co-incubation prevented depolarization. However, the MMP values resulting from these co-incubations were not significantly different when compared to those produced by GW-405833 (10 μM) alone. This lack of significant attenuation by CBR ligand co-incubation relative to GW-405833 (10 μM) alone was not unexpected, given that 10 μM of rimonabant and AM-251 also reduced MMP when tested alone (Figure 9A).

In summary, ligands with high affinity for CBRs expressed in DU-145 cells significantly lowered mitochondrial membrane potential, indicating that these compounds produce mitochondrial injury that potentially contributes to the cell death observed in previous experiments examining LDH release and ATP-dependent cell viability.

## 3. Discussion

The current study confirms and expands on our recently reported findings in Ewing sarcoma (a form of pediatric bone cancer) [33], namely, that several additional cancer types, derived from mouse (C6-glioma), rat (PC-12) and human (HeLa, PC-3, DU-145) origins, similarly express CBRs exhibiting atypical receptor binding and signaling properties relative to canonical CB1 and/or CB2 receptors. Importantly, cannabinoid ligands exhibiting high affinity for these non-canonical CBRs induce cytotoxicity and mitochondrial injury in prostate cancer cells. Therefore, such non-canonical CBRs might potentially serve as a common therapeutic target for drug development of new anti-cancer agents to treat a variety of cancer types. A more complete understanding of the binding and signaling characteristics of these atypical CBRs might also help to resolve many reported discrepancies in the literature regarding whether cytotoxic effects of CBR ligands are mediated via CBR-dependent or -independent mechanisms. Most significantly, future studies are warranted to more clearly define the underlying molecular mechanisms, signaling properties, and cytotoxic profile of these receptors, in order to establish a new class of potentially efficacious anti-cancer agents with reduced adverse effects.

Prostate cancer is the sixth leading cause of cancer death in men globally [54]. Unfortunately, many severe side effects and complications are associated with current treatment options for prostate cancer, including chemo- and radiotherapy, thereby compromising the quality of life for patients [3,8,11]. Our observations that prostate cancer DU-145 and PC-3 cells express non-canonical CBRs, suggest that these potentially novel receptors might be attractive targets for developing ligands eliciting cytotoxicity without the deleterious effects associated with current therapeutic options. Indeed, several studies have been published demonstrating that cannabinoids exhibit efficacy for treating a number of types of cancer, including prostate cancer [55]. When cytotoxic mechanisms of cannabinnoids are investigated, some reports offer evidence of the direct involvement of CBRs in mediating cancer cell death, whereas others suggest that cytotoxic actions are CBR-independent. For example, one recent study shows that WIN-55,212-2 produces anti-tumor effects in prostate cancer mainly through action at CB2 receptors [56]. Conversely, another report suggests that induction of cytotoxicity by WIN-55,212-2 in prostate cancer appears to be independent of CBR involvement [57]. GW-405833, a CB2-selective cannabinoid examined in this study, has been reported previously by others to impact expression levels of proteins involved in energy metabolism and cell growth in pancreatic adenocarcinoma, resulting in cell death [58]. Additionally, inhibition of pancreatic cancer cell growth by GW-405833 has been reported to occur in a CBR-dependent manner resulting in ROS formation and autophagy [59].

Given the unique binding and signaling properties of the non-canonical CBRs characterized in the present study, it could be argued that such receptors might represent splice variants of canonical CB1Rs (e.g., CB1Ra and CB1Rb [60]) or CB2Rs (e.g., Q63R and H316Y splice variants of CB2Rs [61,62]). However, this is unlikely, given that the ligand binding and signaling profile of CBRs detected in the present study is distinct from that of either canonical or CBR splice variants. For example, CP-55,940 does not bind to non-canonical CBRs expressed in all cancer cells examined here, whereas CP-55,940 exhibits high affinity for canonical CBRs and associated splice variants [60]. Furthermore, CP-55,940 and WIN-55,212-2 activate G_i/o_ proteins via canonical CBRs and associated splice variants which then proceed to subsequently inhibit adenylyl cyclase activity [63]. In contrast, in DU-145 and PC-3 cancer cell lines examined in the present study, WIN-55,212-2 failed to increase G_i/o_ protein activity (in membranes) or reduce intracellular cAMP levels (in intact whole cells). Therefore, based on distinct binding and signaling profiles, it is unlikely that CBRs expressed in prostate cancer cells are either canonical CBRs or splice variants of CBRs.

Cannabinoid ligands exhibit affinity for receptors other than canonical CBRs and associated splice variants that might also potentially represent the non-canonical CBRs detected in cancer cells in the present study. One example is the aminoalkyl indole (AI) receptor, a GPCR which has been shown to inhibit microglial cell invasion and growth upon WIN-55,212-2 binding [64]. When bound by agonists, AI receptors activate the G_s_ subtype of G proteins in microglia, enhancing adenylyl cyclase activity to produce an increase in intracellular cAMP [64,65,66]. However, in the present study, when CBR receptors expressed in prostate cancer cells were bound by cannabinoid ligands, no increase in cAMP was observed, and, in fact, data indicate these receptors are likely G_i/o_-linked. Two other potential receptors that could represent CBRs expressed in cancer cells are GPR55 and GPR18 [67]. This is also unlikely, given that both of these orphan GPCRs possess a signaling profile that differs from non-canonical CBRs detected in prostate cancer cells. For example, AM-251 acts as an agonist at GPR55 [67,68] and a partial agonist on GPR18 receptor [69], whereas in our study AM-251 exhibits only antagonist/inverse agonist properties in PC-3 and DU-145 cells. Similarly, while both Δ^9^-THC and CP-55,940 act as full agonists at both GPR18 and GPR55 [67,68,70], both of these cannabinoid ligands are unable to bind non-canonical CBRs expressed in all the cancer cells investigated. However, given that [^3^H]WIN-55,212-2 does not bind to GPR55 and more complete characterization of [^3^H]CP-55,940 binding in these cells is required, participation of GPR55 cannot be completely ruled out. Taken collectively, the unique binding and functional characteristics of CBRs expressed in PC-3 and DU-145 cells, compared to those of other closely related receptors, such as AI, GPR55, or GPR18, suggest that these receptors may instead represent a novel non-canonical CBR that is commonly expressed across many cancer types.

In the present study, several cannabinoids examined (WIN-55,212-2, rimonabant, and AM-251) acted as inverse agonists to decrease [^35^S]GTPγS binding below basal levels but did not increase intracellular cAMP levels in whole cells (also characteristic of inverse agonists). This lack of concordance between signaling assays might be explained by differences in the assays conducted. G protein activation was conducted using membrane homogenates, while modulation of adenylyl cyclase was assessed in whole cells. It is thus possible that compartmentalization of signaling components might contribute to the observed assay differences. For example, CBRs and G_i/o_ proteins might form signaling complexes that are compartmentalized away from adenylyl cyclase enzymes. Additionally, since modulation of adenylyl cyclase modulation occurs downstream from G_i/o_ protein activation [71], it is also possible that the amount of G proteins activated by ligand binding was insufficient to reach a threshold required for modulation of adenylyl cyclase to be observed.

Conflicting evidence has been presented concerning whether CBR upregulation protects against or promotes tumor growth. For example, some reports indicate that high CBR expression is linked to poor prognosis and negative therapeutic outcomes [72,73]. Conversely, other studies suggest that loss of CB1 or CB2 receptors is pro-tumorigenic [74]. Interestingly, findings presented here show that chronic exposure (48 h) of DU-145 and PC-3 cells to both canonical CBR agonists (WIN-55-212-2, GW-405833) and antagonists/inverse agonists (rimonabant, AM-251) induced significant decreases in the expression of non-canonical CBRs (e.g., receptor downregulation). Although not statistically significant, the degree of downregulation produced by the agonists WIN-55,212-2 and GW-405833 tended to be greater than that resulting from prolonged incubation with the antagonists/inverse agonists rimonabant and AM-251. In any case, it is quite unusual for antagonists/inverse agonists to produce receptor downregulation of GPCRs, and these results provide one more piece of evidence that CBRs expressed in prostate cancer cells respond differently to chronic treatment to cannabinoids than canonical CBRs. Importantly, studies have shown that both CB1 and CB2Rs are overexpressed in cancer tissues, and a strong correlation may exist between CBR upregulation and poor prognosis in human tumors in general [75] and in prostate cancer in particular [56]. For example, overexpression of CB2Rs in prostate cancer has been reported to be linked to enhanced cancer cell proliferation, migration, and invasion [76]. These observations are in line with qRT-PCR findings presented in the present study demonstrating significant CB2R mRNA upregulation in both PC-3 and DU-145 cells. Therefore, it is possible that downregulation of non-canonical CBRs expressed in prostate cancer cells (and other types of cancer as well) produced by chronic treatment with cannabinoids might ultimately be shown to produce positive therapeutic outcomes.

In the present study, cell death produced by cannabinoid ligands, as assessed by ATP-dependent cell viability and LDH release, was in general agreement when considering actions of GW-405833 and rimonabant but not WIN-55-212-2 and AM-251. For example, all four cannabinoids reduced ATP-dependent viability, but only GW-405833 and rimonabant produced significant LDH release. One potential explanation might be that drug-induced reductions in intracellular ATP levels might not only reflect cell viability but also cells that are merely metabolically inactive or senescent. In support of this suggestion, it has been reported that ATP generation by oxidative phosphorylation diminishes during senescence due to continuous oncogenic stress [77]. Accordingly, under the conditions tested, cells treated with WIN-55-212-2 and AM-251 could perhaps be metabolically inactive, requiring longer incubation periods to observe more pronounced declines in ATP activity, producing cell death.

Importantly, all cannabinoids examined in this study also decreased mitochondrial membrane potential (MMP), indicative of depolarization. Although speculative at this point, we hypothesize that MMP depolarization might constitute a potential contributing factor to the mechanism of action for cannabinoid-induced cell death observed in the present study. Specifically, we propose that MMP depolarization be considered an early marker of cell death, preceding any decline in ATP activity or increase in LDH release. If so, this might explain the depolarizing effects observed with lower concentrations of cannabinoids, despite an absence of cell death reflected in both LDH release and ATP-dependent viability using these same concentrations.

Finally, the cytotoxic effects of GW-405833 in DU-145 cells as measured by LDH release and reductions in ATP and MMP depolarization were all antagonized by co-incubation with rimonabant and AM-251. This is not surprising, given that both rimonabant and AM-251 exhibited partial “agonist” effects in these assays, relative to the full “agonist” effects produced by GW-405833. As a potential mechanism contributing to this antagonistic effect, it should be noted that rimonabant and AM-251 acted as inverse agonists in the G protein assays (reducing G protein activity below baseline levels), whereas GW-405833 produced no effect of G protein modulation (indicative of a neutral antagonist). However, and very interestingly, AM-630 (a CB2-selective inverse agonist at canonical CBRs), while exhibiting no affinity for the non-canonical CBRs expressed in DU-145 cells, also antagonized cytotoxicity and mitochondrial effects produced by GW-405833 (a CB2-selective agonist at canonical CBRs). Although currently unclear, it is possible that AM-630 binds to an allosteric site on non-canonical CBRs expressed in DU-145 cells that is distinct from the orthosteric site to which [^3^H]WIN-55,212-2 binds to modulate function of these novel receptors (see the following paragraph). In any case, collectively these co-incubation studies indicate that the high affinity CBR ligands examined in this study produce significant cell death of DU-145 prostate cancer cells that is mediated via a common non-canonical CBR receptor.

A potential mechanism contributing to atypical binding and functional characteristics observed in this study could result from heteromerization of CBRs. CB1 and CB2 receptors are members of the class A rhopsodin-like G protein-coupled receptor (GPCR) family [78]. Not only do GPCRs exist individually, they also form monomers and heteromers with other receptors in a process referred to as heteromerization [79]. Heteromerization is observed when two or more GPCRs in proximity interact with each other to form dimers or oligomers that exhibit novel functional and biochemical properties often dissimilar to any of the individual receptors making up the heteromers [80]. Additionally, heterodimers exhibit distinct functional signaling characteristics relative to the properties of individual canonical receptors [81].

It has been reported that CB1 receptors form homomers [82] or heteromers with GPR55 [83]. CB1–CB2 heteromers have also been observed in transfected neuronal cells, neuroblastoma cells, and in several rat brain regions, and, when bound by CB1 or CB2 antagonists, bidirectional negative crosstalk has been observed between both receptors (cross-antagonism) [84]. These observations indicate that heteromer formation results in a novel receptor with unique functional characteristics. Finally, the co-activation of CB1–CB2 receptor heteromers with CB1 or CB2 agonists leads to negative crosstalk reflected in reduced Akt phosphorylation and neurite outgrowth [84].

CB2 receptors also form heteromers via interaction with the GPCR chemokine CXCR4 receptors in both human prostate and breast cancer cells [26,27]. CXCR4 receptor activation plays a key role in controlling cell proliferation and migration, contributing to prostate cancer metastasis. However, and very importantly, when agonists for both CXCR4 and CB2 receptors are administered simultaneously, heteromers are formed, and CXCR4-mediated cell migration of breast cancer cells is reduced relative to CXCR4 activation alone [27]. Similarly, the presence of CB2-CXCR4 heteromers results in a reduction in Ga13/RhoA signaling, leading to inhibition of cell migration and invasion in prostate cancer [26]. Therefore, CB2-CXCR4 heteromers could represent a novel therapeutic target for the inhibition of prostate and breast cancer progression and metastasis.

Such CBR heterodimerization with other receptors in the cancer cell lines examined in this study might not only help to explain the distinct pharmacological and biochemical properties of the non-canonical CBRs observed, but most importantly, it might be anticipated that drugs could be developed that bind heteromers in cancer cells (to promote cell death) without affecting individual canonical receptors expressed in normal healthy cells.

## 4. Materials and Methods

### 4.1. Materials

CP-55,940, 3-isobutyl-1-methylxanthine (IBMX), and nafoxidine hydrochloride were obtained from Sigma-Aldrich (St. Louis, MO). WIN-55,212-2, WIN-55,212-3 (mesylate), etoposide, docetaxel, cannabidiol (CBD), ospemifene, MAM-2201, rimonabant, GW-405833, capsaicin, PDDHV, CAY-10448, HC-030031, HC-067047, and N-oleoyl-valine were all procured from the Cayman Chemical Company (Ann Arbor, MI, USA). AM-630 and AM-251 were acquired from Tocris Bioscience (Bristol, United Kingdom). Radioligands [^3^H]CP-55,940 (164.9 Ci/mmol) and [^35^S]GTPγS (1250 Ci/mmol) were purchased from PerkinElmer (Waltham, MA, USA); [^3^H]WIN-55,212-2 (40 Ci/mmol) was obtained from American Radiolabeled Chemicals (St. Louis, MO, USA); [^3^H]adenine (10 Ci/mmol) was purchased from ViTrax (Placentia, CA, USA). Forskolin was procured from Millipore Sigma (St. Louis, MO, USA). ACEA, MAM-2201, 5F-AKB48, FDU-PB-22, 5F-AB-PINACA, and 5F-AKB-48 were generously provided by William E. Fantegrossi, Ph.D. (University of Arkansas for Medical Sciences, Little Rock, AR, USA) through the Drug Enforcement Administration (DEA) Special Testing and Research Laboratory program. The LDH cytotoxicity kit was purchased from Biovision (Milpitas, CA, USA). All drugs were dissolved in 100% DMSO to obtain a 10 mM stock solution, divided into aliquots, and kept at −20 °C until use. All additional reagents were purchased from Fisher Scientific Inc. (Hampton, NH, USA).

### 4.2. Cell Culture

PC-12 pheochromocytoma and C6-glioma cells were purchased from the American Type Culture Collection (ATCC; Manassas, VA, USA). DU-145 and PC-3 prostate cancer cells and immortalized pancreatic NPrEC cells [51] were generously provided by Yuet-Kin Leung, Ph.D. (University of Arkansas for Medical Sciences, Little Rock, AR, USA). Chinese hamster ovary (CHO) cells stably transfected with wild-type recombinant cannabinoid type-1 receptors (hCB1) and designated as CHO-hCB1 were purchased from DiscoverRx Corporation (Fremont, CA, USA). Chinese hamster ovary (CHO) cells stably transfected with wild-type recombinant human cannabinoid type-2 receptors (hCB2) and designated as CHO-hCB2 were produced in our laboratory [85]. DU-145, PC-12, and C6-glioma cells were all cultured in Dulbecco’s Modified Eagle’s Medium (DMEM) purchased from Corning Life Sciences (Tewksbury, MA, USA). PC-3 and CHO-hCB1 cells were cultured in HAM’s F-12 K (Kaighn’s) media (Thermo Fisher Scientific; Waltham, MA, USA). HeLa cervical cancer cells were provided as a gift from Vladimir Lupashin, Ph.D. (University of Arkansas for Medical Sciences, Little Rock, AR, USA) and cultured in Minimum Essential Medium Eagle Medium (MEM) obtained from Sigma-Aldrich (St. Louis, MO). For all cells, 10% FetalPlex animal serum complex (Gemini Bio Products; West Sacramento, CA, USA) and 1% penicillin/streptomycin (10,000 IU/mL penicillin, 10,000 μg/mL streptomycin; Invitrogen, Carlsbad, CA, USA) were added to the culture media. Culture medium for CHO-hCB1 and CHO-hCB2 cells additionally contained 0.5% of the G-418 selection antibiotic (Sigma-Aldrich; St. Louis, MO, USA). NPrEC cells were maintained in a keratinocyte serum-free medium (KSFM; Life Technologies, Carlsbad, CA, USA). Fetalplex was heat-shocked prior to use for all cells except HeLa. All cell types were cultured in a humidified incubator maintained at 37 °C with 5% CO_2_. Cells were used between passages 10–26. Cells were detached for harvesting and collected using trypsin-EDTA (0.25%) (Thermo Fisher Scientific; Waltham, MA, USA) upon reaching 90–95% confluency. All cells were then either frozen as pellets at −80 °C for membrane preparation, seeded for assessment of cell viability and mitochondrial integrity, or reseeded into flasks for continuous culturing.

### 4.3. Quantitative Real-Time PCR (qRT-PCR)

Total RNA from DU-145 and PC-3 cells was extracted by Qiagen RNeasy kit (Qiagen; Venlo, The Netherlands). Human prostate RNA served as a positive control (Thermo Fisher Scientific; Waltham, MA, USA). cDNA was then synthesized by reverse transcription of total RNA at 50 °C for 50 min using SuperScript III Reverse Transcriptase kit (Thermo Fisher Scientific; Waltham, MA, USA) and maintained at 4 °C, as detailed previously [86]. Real-time PCR reactions were prepared using Applied BiosystemsTM TaqManTM Universal Master Mix II with uracil-N-glycosylase (UNG) (Thermo Fisher Scientific; Waltham, MA, USA). qRT-PCR assays were conducted using an ABI PRISM 7000 (Applied Biosystems; Waltham, MA, USA) by activating AmpliTaq Gold DNA polymerase initially at 95 °C for 10 min, followed by denaturation for 40 cycles at 95 °C for 15 s, and then annealing and extension at 60 °C for 1 min. Relative mRNA levels for each sample were normalized to GAPDH and calculated in terms of Ct (the amplification cycle threshold). All Taqman real-time PCR primers, including for hCB1 (Assay ID: Hs01038522_s1), hCB2 (Assay ID: Hs05019229_s1), and GAPDH (Assay ID: Hs02786624_g1), were obtained from Thermo Fisher Scientific (Waltham, MA, USA). Data analysis was carried out using the 2^−ΔΔCt^ method to obtain fold change. Fold changes are presented as mean values (N = 3 for each treatment group) as described previously [87].

### 4.4. Membrane Preparation

Membranes were prepared as described previously [88]. Briefly, frozen pellets of all cell lines were thawed on ice and suspended in ice-cold buffer consisting of 3 mM MgCl_2_, 1 mM EDTA, and 50 mM HEPES (pH 7.4). Suspended pellets were then homogenized 10 times using a 40 mL Dounce glass homogenizer and centrifuged at 40,000× *g* for 10 min at 4 °C. Supernatants were aspirated and removed, and the homogenization process was repeated three times. Lastly, pellets were suspended in ice-cold 50 mM HEPES (pH 7.4), divided into aliquots, and frozen at −80 °C. The protein concentration of crude membrane homogenates was determined using the BCA™ Protein Assay (Thermo Scientific; Rockford IL, USA).

### 4.5. Competition Receptor Binding Assays

#### 4.5.1. Cancer Cell Line Screening for the Presence of CBRs

Competition receptor binding screens were first carried out to investigate whether CBRs were present in cancer cell lines examined, using well-established non-selective CB1/CB2 receptor radioligands, [^3^H]CP-55,940 and [^3^H]WIN-55,212-2. The assay was conducted as previously detailed [33]. First, membrane homogenates prepared from CHO-hCB1 (100 μg), CHO-hCB2 (50 μg), mouse brain (25 μg), C6-glioma (200 μg), PC-12 (200 μg), HeLa cells (200 μg), PC-3 (200 μg), or DU-145 cells (100 μg) incubated with 0.2 nM [^3^H]CP-55,940 or 1 nM [^3^H]WIN-55,212-2 in the absence or presence of 1 μM of non-radioactive CP-55,940 or WIN-55,212-2 (a receptor-saturating concentration for canonical CB1 and CB1 receptors) in an incubation buffer containing 5 mM MgCl_2_, 50 mM Tris–HCl buffer (pH 7.4), and 0.05% bovine serum albumin. The amount of membrane homogenates of each cell line used in the receptor binding experiments was determined by initial optimization experiments examining [^3^H]CP-55,940 and [^3^H]WIN-55,212-2 receptor binding in samples containing membrane amounts ranging from 50 to 500 μg per assay tube (data not shown). All binding reactions were conducted in triplicate, in a final volume of 1 mL, and, following mixing, were incubated for 20 min in a 37 °C water bath to achieve equilibrium. After incubation, reactions were terminated by rapid vacuum filtration through Whatman GF/B glass fiber filters (Brandel; Gaithersburg, MD, USA), followed by three washes of 4 mls of ice-cold filtration buffer (50 mM Tris, pH 7.4, and 0.05% bovine serum albumin). Samples were punched out from filter papers into scintillation vials, to which 4 mls of ScintiverseTM-BDR cocktail scintillation fluid (Fisher Scientific; Hampton, NH, USA) was added. Filters were incubated at room temperature for 24 h in scintillation fluid, vortexed, and radioactivity (measured as DPMs) was quantified via a liquid scintillation spectrophotometer (Tri Carb 2100 TR Liquid Scintillation Analyzer, Packard Instruments, Meriden, CT, USA).

#### 4.5.2. Homologous Receptor Binding Assays

Homologous receptor binding assays were performed as previously reported [33], to determine the density (B_MAX_) of CBRs and affinity (K_D_) of WIN-55,212-2 for expressed receptors in all cancer cell lines examined. Similar to competition receptor binding assays, membranes prepared from either mouse brain (25 μg), C6-glioma (200 μg), PC-12 (200 μg), HeLa cells (200 μg), PC-3 (200 μg), or DU-145 cells (100 μg) were incubated with increasing concentrations (10^−11^ to 10^−6^ M) of non-radioactive WIN-55,212-2 or vehicle and a single concentration (1 nM) of [^3^H]WIN-55,212-2 in incubation buffer (5 mM MgCl_2_, 50 mM Tris–HCl buffer, pH 7.4 with 0.05% bovine serum albumin). Non-specific binding was defined as radioactivity remaining in the presence of 10 μM of non-radioactive WIN-55,212-2 and subtracted from total binding to determine specific [^3^H]WIN-55,212-2 binding. All binding reactions were conducted in triplicate, in a final volume of 1 mL, and, following mixing, were incubated for 20 min in a 37 °C water bath to achieve equilibrium. After incubation, reactions were terminated by rapid vacuum filtration and processed for radioactivity, as detailed in Section 4.5.1.

#### 4.5.3. Screening for Binding of Known Cannabinoid Ligands to Expressed CBRs in Prostate Cancer Cells

These experiments were conducted to screen well-characterized cannabinoid ligands with high affinity for canonical CB1 and/or CB2Rs for binding to CBRs expressed in prostate cancer cells lines, as detailed in [33]. Briefly, membrane homogenates of DU-145 (100 μg) and PC-3 (200 μg) cells were incubated with a single concentration (1 μM; a receptor-saturating concentration of all compounds for canonical CB1 and CB1 receptors) of 15 different CBR ligands and [^3^H]WIN-55,212-2 (1 nM) in incubation buffer (5 mM MgCl_2_, 50 mM Tris–HCl buffer, pH 7.4, with 0.05% bovine serum albumin). Non-specific binding was defined as radioactivity remaining in the presence of 10 μM of non-radioactive WIN-55,212-2 and subtracted from total binding to determine specific [^3^H]WIN-55,212-2 binding. All binding reactions were conducted in triplicate, in a final volume of 1 mL, and, following mixing, were incubated for 20 min in a 37 °C water bath to achieve equilibrium. After incubation, reactions were terminated by rapid vacuum filtration and processed for radioactivity, as detailed in Section 4.5.1.

#### 4.5.4. Affinity (K_i_) Determination of Cannabinoid Ligands for Expressed CBRs in Prostate Cancer Cells

The assay was performed as previously detailed [33]. Homogenates prepared from prostate cancer cells (DU-145, 100 μg and PC-3, 200 μg) were incubated with increasing concentrations (10^−10^ to 10^−5^ M) of rimonabant, AM-251, and GW-405033, and 1 nM of [^3^H]WIN-55,212-2 in incubation buffer (5 mM MgCl_2_, 50 mM Tris–HCl buffer, pH 7.4, with 0.05% bovine serum albumin). Non-specific binding was defined as radioactivity remaining in the presence of 10 μM of non-radioactive WIN-55,212-2 and was subtracted from total binding to determine specific [^3^H]WIN-55,212-2 binding. All binding reactions were conducted in triplicate, in a final volume of 1 mL, and, following mixing, were incubated for 20 min in a 37 °C water bath to achieve equilibrium. After incubation, reactions were terminated by rapid vacuum filtration and processed for radioactivity, as detailed in Section 4.5.1.

### 4.6. Modulation of [^35^S]GTPγS Binding by Cannabinoid Ligands in Prostate Cancer Cells

[^35^S]GTPγS binding experiments were conducted to examine G protein modulation by cannabinoids in prostate cancer cell lines DU-145 and PC-3, as detailed previously [33]. Briefly, CHO-hCB2 (50 μg), PC3 (100 μg), or DU-145 (100 μg) membrane homogenates were incubated with a single receptor-saturating concentration (10^−5^ M) of cannabinoid ligands to be examined and 0.1 nM [^35^S]GTPγS in incubation buffer (20 mM HEPES buffer, pH 7.4, 10 μM GDP, 100 mM NaCl, 0.1% bovine serum albumin, and 10 mM MgCl_2_). Non-specific binding was defined as radioactivity remaining in the presence of 10 μM of non-radioactive GTPγS and subtracted from total binding to determine specific [^35^S]GTPγS binding. All binding reactions were conducted in triplicate, in a final volume of 1 mL, and, following mixing, were incubated for 30 min at 30 °C. After incubation, reactions were terminated by rapid vacuum filtration through Whatman GF/B glass fiber filters (Brandel; Gaithersburg, MD, USA), followed by three washes of 4 mls of ice-cold filtration buffer (50 mM Tris, pH 7.4, and 0.05% bovine serum albumin). Samples were punched out from filter papers into scintillation vials, to which 4 mls of ScintiverseTM-BDR cocktail scintillation fluid (Fisher Scientific; Hampton, NH, USA) was added. Filters were incubated at room temperature for 24 h in scintillation fluid, vortexed, and radioactivity (measured as DPMs) was determined via a liquid scintillation spectrophotometer (Tri Carb 2100 TR Liquid Scintillation Analyzer, Packard Instruments, Meriden, CT, USA).

### 4.7. Modulation of Adenylyl Cyclase Activity by Cannabinoid Ligands in Prostate Cancer Cells

These experiments were conducted to examine modulation of adenylyl cyclase activity by cannabinoid ligands in prostate cancer cell lines DU-145 and PC-3, as described earlier [33]. CHO-hCB2, DU-145, or PC-3 cells were seeded into 24 well plates at a density of 8 × 10^6^ cells per plate in growth media (DMEM for DU-145 and CHO-hCB2 cells or F12-K for PC-3 cells). Growth media contained FetalPlex (10%) and 1% pennicillin/streptomycin. Cells were cultured overnight in a humidified incubator maintained at 37 °C with 5% CO_2_. For some experiments, a subset of wells were treated overnight with pertussis toxin (2 μL/mL) to abolish G_i_/G_o_ protein-mediated signaling. Following overnight incubation, growth media was replaced with an incubation media containing either DMEM (for DU-145 and CHO-hCB2) or F12-K (for PC-3) with 0.9 g/l NaCl, 2.5 μCi/mL of [^3^H]adenine, and 0.5 mM IBMX, and cells were kept in a humidified incubator maintained at 37 °C with 5% CO_2_ for 4 h. After [^3^H]adenine incubation, media was replaced with 0.5 mL of a Krebs–Ringer–HEPES solution (10 mM HEPES, 110 mM NaCl, 25 mM Glucose, 55 mM Sucrose, 5 mM KCl, 1 mM MgCl_2_, and 1.8 mM CaCl_2_ at pH 7.4) containing 0.5 mM IBMX, 10 μM forskolin and a receptor-saturating concentration (10^−5^ M) of cannabinoid ligands to be examined. Plates were then floated in a 37 °C water bath for 15 min, followed by termination of reactions by addition of 50 μL of 2.2 N HCl to each well. Then, [^3^H]cAMP was isolated by alumina column chromatography and collection in a 4 mL elution volume. Finally, 10 mL of Scintiverse^TM^ BD Cocktail scintillation fluid (Fisher Scientific; Hampton, NH, USA) was added to eluates and radioactivity (quantified as DPMs) was quantified via a liquid scintillation spectrophotometer (Tri Carb 2100 TR Liquid Scintillation Analyzer, Packard Instruments, Meriden, CT, USA).

### 4.8. Effect of Chronic Cannabinoid Ligand Treatment on CBR Density in Prostate Cancer Cells

These studies were conducted to determine the effect of chronic exposure of PC-3 and DU-145 cells to cannabinoid ligands with high affinity for non-canonical CBRs on receptor expression. PC-3 or DU-145 cells were seeded into T25 flasks at a density of 1.6 × 10^6^ cells in normal medium and cultured at 37 °C with 5% CO_2_ for 24 h. Following overnight seeding, culture media was aspirated and replaced with serum-free medium containing vehicle or a receptor-saturating concentration (10 μM) of each cannabinoid ligand to be tested. Cells were subsequently cultured at 37 °C with 5% CO_2_ for an additional 48 h. After chronic drug exposure, cells were thoroughly washed three times with warmed serum-free and drug-free media to remove residual drug and then harvested using trypsin–EDTA (0.25%). Cells were then centrifuged at 1200 rpm for 10 min, and pellets were stored in −80 °C until future use.

Upon thawing, 150 μg of cell lysates resuspended in ice-cold binding buffer (3 mM MgCl_2_, 1 mM EDTA, and 50 mM HEPES, pH 7.4) were incubated with [^3^H]WIN-55,212-2 (1 nM) a receptor-saturating concentration of non-radioactive WIN-55,212-2 (10 μM) to determine specific [^3^H]WIN-55,212-2 binding. All binding reactions were conducted in triplicate, in a final volume of 1 mL, and following mixing were incubated for 20 min in a 37 °C water bath to achieve equilibrium. After incubation, reactions were terminated by rapid vacuum filtration through Whatman GF/B glass fiber filters (Brandel; Gaithersburg, MD, USA), followed by three washes of 4 mls of ice-cold filtration buffer (50 mM Tris, pH 7.4, and 0.05% bovine serum albumin). Samples were punched out from filter papers into scintillation vials, to which 4 mls of ScintiverseTM-BDR cocktail scintillation fluid (Fisher Scientific; Hampton, NH, USA) was added. Filters were incubated at room temperature for 24 h in scintillation fluid, vortexed, and radioactivity (measured as DPMs) was quantified via a liquid scintillation spectrophotometer (Tri Carb 2100 TR Liquid Scintillation Analyzer, Packard Instruments, Meriden, CT, USA).

### 4.9. Effect of Cannabinoid Ligands on Cytotoxicity in DU-145 Prostate Cancer Cells: LDH Release

DU-145 cytotoxicity (cell death) was assessed as previously described [89] using the LDH-Cytotoxicity Colorimetric Assay Kit II (BioVision Research Products; Milpitas, CA USA, #K313) to quantify lactate dehydrogenase (LDH) release from damaged or dying cells. DU-145 cells were seeded at a density of 130 × 10^3^ cells/well in 6-well plates and maintained in an incubator at 37 °C with 5% CO_2_ for 24 h. The next day, seeding medium was replaced with serum-free medium containing either vehicle or experimental drugs (10 or 50 μM). Treated cells were incubated for an additional 24 h, followed by quantification of LDH release. LDH released from damaged or dying cells oxidizes lactate to produce NADH which then reacts with a water-soluble tetrazolium salt (WST) reagent to produce a yellow-colored product. The intensity of the yellow-colored product was detected with an OD reading at 450 nm (quantified by a BioTek microplate reader interfaced with SoftMax Pro 5.4.5 software; Winooski, VT, USA) and was directly proportional to the amount of LDH released. Percent cytotoxicity was calculated according to the manufacturer’s protocol as the following:

$$\%\;\mathrm{Cytotoxicity}=\mathrm{OD}450\;\left(\frac{\mathrm{Supernatant}}{\mathrm{Supernatant}+\mathrm{Lysate}}\right)$$

### 4.10. Effect of Cannabinoid Ligands on ATP-Dependent Cell Viability of DU-145 Prostate Cancer Cells

Adenosine 5′-triphosphate (ATP) levels were quantified as a measure of DU-145 cell viability in response to cannabinoid treatement using an ATP-luciferase-based bioluminescence assay kit (CellTiter-Glo^®^, Sigma, MO, USA) and BioTek microplate reader (Winooski, VT, USA). DU-145 cells were seeded at a density of 8 × 10^3^ cells/well in a 96-well plate and incubated for 24 h at 37 °C with 5% CO_2_. The next day, cells were treated with either vehicle or experimental drugs (10 or 50 μM) and incubated for another 24 h at 37 °C with 5% CO_2_. ATP levels were assessed following incubation by interpolation from an ATP standard curve according to the manufacturer’s protocol, as described previously [90].

### 4.11. Effect of Cannabinoid Ligands on Mitochondrial Membrane Potential in DU-145 Prostate Cancer Cells: JC-1 Staining

Mitochondrial membrane potential (MMP) following cannabinoid ligand exposure was assessed by 5,5′,6,6′-tetrachloro-1,1′,3,3′-tetraethylbenzimidazol-carbocyanine iodide (JC-1) staining as described previously [89]. Briefly, DU-145 cells were seeded at a density of 30 × 10^3^ cells/well in 24-well plates and maintained in an incubator at 37 °C with 5% CO_2_ overnight. The next day, cells were treated with either vehicle or experimental drugs (10 or 50 μM) and incubated for another 24 h at 37 °C with 5% CO_2_. JC-1was then added to each well to achieve a final concentration of 7.5 μM and incubated at 37 °C for an additional 30 min. Cells were washed with JC-1 dye buffer (C2006-3, Beyotime Institute of Biotechnology, Haiman, Jiangsu, China) and analyzed using a BioTek microplate reader interfaced with SoftMax Pro 5.4.5 software (Winooski, VT, USA) to obtain OD readings at 525 nm excitation and 590 nm emission.

### 4.12. Statistical Analyses

Curve-fitting and all statistical analyses were performed using GraphPad Prism^®^ v8.0g (GraphPad Software, Inc.; San Diego, CA, USA). For qRT-PCR experiments, a one-way ANOVA, followed by Dunnett’s test as a post hoc test was utilized to compare fold change of PC-3 and DU-145 cancer cells to an arbitrary value of 1, set to the amount of mRNA transcribed from a human normal prostate control. For CBR screening studies, a one-sample *t*-test was performed to determine differences from 100% (e.g., to quantify compounds producing displacement), followed by a one-way ANOVA and Tukey’s post hoc tests to compare displacement produced by all drugs. For homologous receptor binding assays, curve-fitting and statistical analyses were carried out using non-linear regression via a one-site homologous competition binding equation to determine the affinity (K_D_) of [^3^H]WIN-55,212-2 for CBRs and CBR density (B_MAX_) in membrane homogenates for all cancer cells investigated. To compare specific CBR affinity (K_i_) between ligands, non-linear regression analysis was employed to determine experimental IC_50_ values from competition receptor binding assays, which were then converted to K_i_ values using the Cheng–Prusoff equation [35]. K_i_ values were converted to pK_i_ values so that parametric tests could be used and statistically compared by employing a one-way ANOVA followed by Tukey’s post hoc test. For statistical analyses of [^35^S]GTPγS, adenylyl cyclase, and chronic experiments, a one-sample *t*-test was performed to detect differences from 100% (e.g., basal levels), followed by comparison of all means by a one-way ANOVA and Tukey’s post hoc test. For LDH release, ATP-dependent cell viability and JC-1 staining assays, drug effects were first analyzed by a one-sample *t*-test to determine differences from 0 or 100% (vehicle), followed by a one-way ANOVA and Dunnett’s post hoc test to compare all means. Significance for all statistical tests was defined at a minimal level of *p* < 0.05.

## 5. Conclusions

Collectively, the findings reported in this study indicate that a broad range of cancer cell types, including prostate cancer cells, express common CBRs with unique ligand binding and signaling profiles relative to canonical CBRs. Specifically, cannabinoid ligands exhibiting high affinity for expressed non-canonical CBRs in prostate cancer cells induced cytotoxicity. Therefore, expressed non-canonical CBRs might potentially represent a therapeutic target to slow tumor growth. Moreover, developing selective ligands for and identification of signaling pathways of such non-canonical cannabinoid receptors may provide novel, safe, and efficacious therapeutic options for treating various cancer types.

## Figures and Tables

**Table 1:** CBR affinity (K_i_) of tested compounds in PC3 and DU-145 membranes.

[^3^H]WIN-55,212-2 (1 nM) Competition Binding						
	*PC-3*			*DU-145*		
Drug	K_i_ (nM)	pK_i_ (−log K_i_)	N	K_i_ (nM)	pK_i_ (−log K_i_)	N
Rimonabant	54.1	8.01 ± 0.08 ^a^	4	102	7.04 ± 0.15 ^a^	3
AM-251	58.7	7.24 ± 0.05 ^a^	3	69.5	7.19 ± 0.02 ^a^	3
GW-405833	296	6.47 ± 0.17 ^b^	5	290	6.5 ± 0.12 ^b^	5

^a,b^ Different letters indicate that pK_i_ values are significantly different from each other (one-way ANOVA followed by Tukey’s post hoc test for multiple comparisons, *p* < 0.05).

## Data Availability

All data were included in the manuscript.

## Abbreviations

2-AG	2-Arachidonoyl Glycerol
CBD	Cannabidiol
CB1R	Cannabinoid Receptor Type-1
CB2R	Cannabinoid Receptor Type-2
CBRs	Cannabinoid Receptors
CBD	Cannabidiol
DMEM	Dulbecco’s Modified Eagle Medium
ECS	Endocannabinoid System
GPCR	G protein-Coupled Receptor
GTPγS	Guanosine 5-O-[gamma-thio] triphosphate
LDH	Lactate Dehydrogenase
MMP	Mitochondrial Membrane Potential
IBMX	Isobutyl-methyl-xanthine
qRT-PCR	Quantitative Real-time Polymerase Chain Reaction
ROS	Reactive Oxygen Species
THC	Δ^9^-Tetrahydrocannabinol
